# Effectiveness of Microecological Preparations for Improving Renal Function and Metabolic Profiles in Patients With Chronic Kidney Disease

**DOI:** 10.3389/fnut.2022.850014

**Published:** 2022-09-12

**Authors:** Jiaxing Tan, Huan Zhou, Jiaxin Deng, Jiantong Sun, Xiaoyuan Zhou, Yi Tang, Wei Qin

**Affiliations:** ^1^Division of Nephrology, Department of Medicine, West China Hospital, Sichuan University, Chengdu, China; ^2^West China School of Medicine, Sichuan University, Chengdu, China; ^3^West China School of Public Health, West China Forth Hospital of Sichuan University, Chengdu, China

**Keywords:** probiotics, prebiotics, synbiotics, chronic kidney disease, renal function, metabolic profiles

## Abstract

**Background:**

Determining whether microecological preparations, including probiotics, prebiotics, and synbiotics, are beneficial for patients with chronic kidney disease (CKD) has been debated. Moreover, determining which preparation has the best effect remains unclear. In this study, we performed a network meta-analysis of randomized clinical trials (RCTs) to address these questions.

**Methods:**

MEDLINE, EMBASE, PubMed, Web of Science, and the Cochrane Central Register of Controlled Trials were searched. Eligible RCTs with patients with CKD who received intervention measures involving probiotics, prebiotics, and/or synbiotics were included. The outcome indicators included changes in renal function, lipid profiles, inflammatory factors, and oxidative stress factors.

**Results:**

Twenty-eight RCTs with 1,373 patients were ultimately included. Probiotics showed greater effect in lowering serum creatinine [mean difference (MD) −0.21, 95% confidence interval (CI) −0.34, −0.09] and triglycerides (MD −9.98, 95% CI −19.47, −0.49) than the placebo, with the largest surface area under the cumulative ranking curve, while prebiotics and synbiotics showed no advantages. Probiotics were also able to reduce malondialdehyde (MDA) (MD −0.54, 95% CI −0.96, −0.13) and increase glutathione (MD 72.86, 95% CI 25.44, 120.29). Prebiotics showed greater efficacy in decreasing high-sensitivity C-reactive protein (MD −2.06, 95% CI −3.79, −0.32) and tumor necrosis factor-α (MD −2.65, 95% CI −3.91, −1.39). Synbiotics showed a partially synergistic function in reducing MDA (MD −0.66, 95% CI −1.23, −0.09) and high-sensitivity C-reactive protein (MD −2.01, 95% CI −3.87, −0.16) and increasing total antioxidant capacity (MD 145.20, 95% CI 9.32, 281.08).

**Conclusion:**

The results indicated that microbial supplements improved renal function and lipid profiles and favorably affected measures of oxidative stress and inflammation in patients with CKD. After thorough consideration, probiotics provide the most comprehensive and beneficial effects for patients with CKD and might be used as the best choice for microecological preparations.

**Systematic Review Registration:**

https://www.crd.york.ac.uk/prospero/display_record.php?ID=CRD42022295497, PROSPERO 2022, identifier: CRD42022295497.

## Introduction

Chronic kidney disease (CKD) is characterized by renal damage with either structural or functional abnormalities for longer than 3 months (1). As a major global health problem, CKD has recently attracted more attention since it could contribute to end-stage renal disease (ESRD) and cardiovascular disease (CVD), with an estimated prevalence of 13.4% (2). Dysbiosis of the gut microbiota causes pathogenic bacteria to increase while beneficial bacterial species decrease, thus proving its role in the progression of ESRD and CVD in patients with CKD (3). Although the exact pathogenesis of the renal-gut axis is not fully understood, gut dysbiosis can lead to an increase in uremic toxins, activation of systemic inflammation and immune system, and metabolic profile disorders, which may participate in its pathogenesis and cause further deterioration of renal function and increased risk of CVD (4).

In this context, microecological preparations, comprising probiotics, prebiotics, and synbiotics, have been proposed as a complementary therapy to restore gut microbial communities (5). Probiotics are live microorganisms that can provide health benefits for individuals. Prebiotics refer to non-living substrates that provide nutrients for beneficial gut microorganisms and promote their growth. Synbiotics are a combination of probiotics and prebiotics (6). Several studies have reported that probiotics, prebiotics, and/or synbiotics are good for the treatment of CKD by modulating the immune response, improving glomerular function, and lowering serum uremic toxins and inflammatory factors. However, some other articles reached contradictory conclusions (7–9). Although a small number of studies have systematically evaluated the efficacy of probiotics, prebiotics, and synbiotics in the treatment of CKD, their conclusions are inconsistent and very different (10–13). Moreover, evidence that directly compares probiotics, prebiotics, and synbiotics and determines which microbial supplement is the best for patients with CKD remains unknown, therefore, preventing clinicians from providing patients with personalized medical care. Consequently, we performed a network meta-analysis to obtain direct and indirect comparative evidence on the effects of probiotics, prebiotics, and synbiotics on renal function and metabolic indicators covering lipid profiles, inflammatory factors, and oxidative stress indices in patients with CKD.

## Methods

This systematic review and network meta-analysis were performed according to the Preferred Reporting Items for Systematic Reviews and Meta-Analyses (PRISMA) extension statement guidelines for network meta-analysis (14). The protocol of the review was submitted to the International Prospective Register of Systematic Reviews (www.crd.york.ac.uk/PROSPERO), and the registration number was CRD42022295497.

### Data Sources and Search Strategy

MEDLINE, EMBASE, PubMed, Web of Science, and the Cochrane Central Register of Controlled Trials (CENTRAL) were searched on August 15, 2021, using a combination of medical subject headings (MeSH) and free text searches, such as “probiotic,” “prebiotic,” “synbiotic,” “chronic kidney disease,” “CKD,” “randomized controlled trial,” or “RCT”. The details of the electronic search strategies are shown in Supplementary Table S1. In addition, some articles in the references of the relevant review and meta-analysis were manually identified. All the references that we searched were well managed using Endnote X9.3.3 (BId 13966) software.

### Study Selection and Inclusion Criteria

Two investigators (ZH and TJX) independently screened the title and abstract; if disagreements occurred, a decision was made through discussion or by a third investigator (QW). A review of the full-text articles was also completed by the two investigators, and the reasons why the articles were included or excluded are recorded explicitly in Supplementary Table S2.

The eligible articles must meet the following criteria: (a) participants: patients with CKD; (b) intervention measures: probiotic, prebiotic, or synbiotic; (c) control measures: placebo; (d) outcomes: changes in renal function or metabolic profiles; and (e) study design: RCT. If more than one study that focused on the same population had different meaningful outcome indicators, all of them were allowed for final analysis.

The studies were excluded if they had the following characteristics: (a) participants with infection, liver disease, heart failure, a history of inflammatory bowel disease (IBD), cancer, autoimmune disease, and acquired immune deficiency syndrome (AIDS), and patients who took antibiotics, immunosuppressants, anti-inflammatory drugs, catabolic drugs, antioxidant vitamin supplements, prebiotics, probiotics, and synbiotics in the 3 months before the start of the studies; (b) the outcomes were unrelated; (c) the study was not an RCT; and (d) the data were unavailable.

### Data Extraction and Quality Assessment

Two researchers (ZH and TJX) separately extracted data from the original literature with an Excel spreadsheet, and a third researcher (QW) was in charge of settling discrepancies. The content of the extraction contained the studies' features (including title, first author, publication year, country, study design, type of participants, intervention, control measurements, and follow-up duration) and the patients' basic demographic characteristics [including sample size, number of males, mean age, and body mass index (BMI)].

The outcomes used in this study mainly included changes in renal function, lipid profiles, oxidative stress indicators, and inflammatory indicators. More precisely, changes in creatinine, estimated glomerular filtration rate (eGFR), total cholesterol (TC), triglycerides (TG), glutathione (GSH), malondialdehyde (MDA), total antioxidant capacity (TAC), high sensitivity C-reactive protein (hs-CRP), interleukin-6 (IL-6), and tumor necrosis factor-α (TNF-α) were analyzed to investigate the efficacy of probiotics, prebiotics, and synbiotics in the treatment of CKD.

The Cochrane Collaboration's tool was used to assess the quality of all the RCTs (15). Two authors (DJX and SJT) independently evaluated the bias (including selection, performance, detection, attrition, and reporting bias), according to the Cochrane Handbook for Systematic Reviews, rating as low risk, high risk, or unclear for each study (16). If disagreements were presented, the third author (ZH) reevaluated the bias. Review Manager (RevMan) (version 5.3.5) was used to draw the figures of bias assessment.

### Data Synthesis and Analysis

To compare the therapeutic effects of different interventions, the mean ± standard deviation (SD) of the change value from the baseline of each indicator was needed. For the RCTs that only had baseline data and last time-point data, a formula was used to approximately calculate the mean ± SD of each change value from baseline. The formula was as follows: SD2 change = SD2 baseline + SD2 final – (2 × correlation coefficient × SD baseline × SD final), and the correlation coefficient (*R*) was recommended to be 0.5 (17).

After data preparation, Stata software (version 13.1, USA) was used to perform the network meta-analysis. The functional program package used to process raw data was the “network” package, which is involved in the “mvmeta” package. Statistical assumptions of similarity, transitivity, and consistency of the data were examined with relevant commands to verify the reliability of the analysis results. Various graphs were drawn *via* the following commands: “networkplot” command was used for drawing network geometry or network plot, “netfunnel” for network funnel plot with random effect model to check publication bias, “intervalplot” for interval plot or forest plot, and “sucra” for treatment rankings and surface area under the cumulative ranking curve (SUCRA) (18). Analysis of heterogeneity mainly relied on subgroup analysis and sensitivity analysis. The treatment effects of probiotics, prebiotics, and synbiotics compared with placebo were expressed as the mean difference (MD) with a 95% confidence interval (CI). The 95% CIs excluding 0 were considered statistically significant.

## Results

### Study Selection and Characteristics

**Figure 1:**
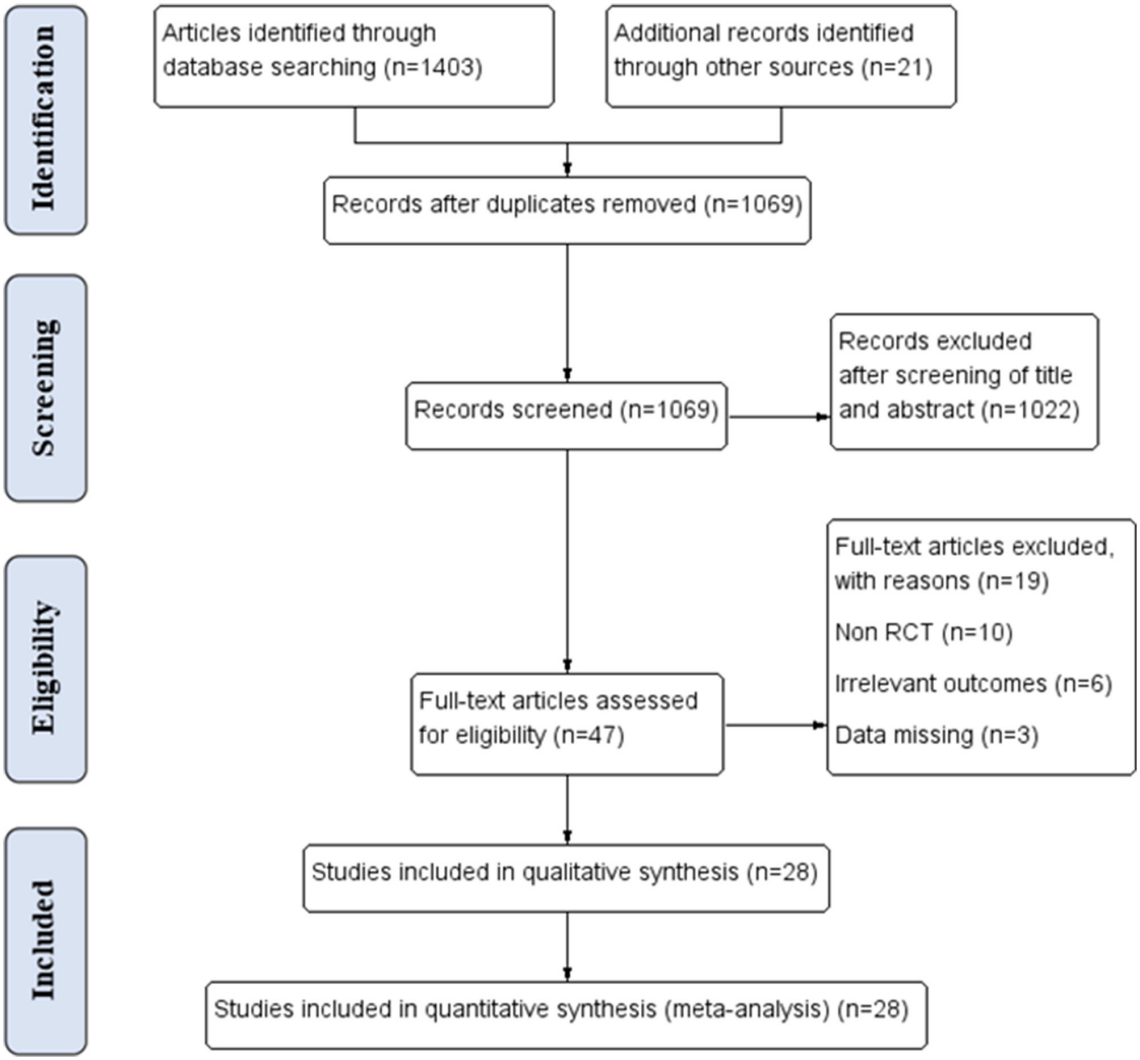
PRISMA flow diagram of the study selection procedure.

A total of 1,403 articles were searched through databases, and an additional 21 records were manually identified. After removing 355 duplicates, 1,069 records were screened by titles and abstracts. The remaining 47 full-text articles were assessed for eligibility, 19 of which were excluded for different reasons. Finally, a total of 28 studies that included 1,373 participants were analyzed in the network meta-analysis (Figure 1). Details of each article are shown in Supplementary Table S3.

The basic characteristics of the 28 RCTs are summarized in Table 1 (7–9, 15, 19–42). Among them, almost all the studies were double-blinded, except for five (2 were single-blinded, 2 were open-label, and the other was triple-blinded). Notably, the participants were not exactly the same in the included studies. The majority of them enrolled patients that were undergoing hemodialysis (15, 53.6%). Four studies included patients with diabetic nephropathy, and 2 RCTs focused on diabetic patients undergoing hemodialysis. Another 4 studies were conducted on patients with CKD from stages 3 to 5. The remaining 3 RCTs included patients with automated peritoneal dialysis (APD), non-diabetic, non-dialysis-dependent CKD (NDD-CKD), and kidney transplant (KTR).

**Table 1:** Basic characteristics of the included studies.

**References**	**Country**	**Type of study**	**Participant**	**Intervention/control**	**Diet restrictions**	**Sample size (I/C)**	**Period (weeks)**	**Male (I/C) (n)**	**Age (years)**	**BMI (kg/m^**2**^)**
Abbasi et al. (19)	Iran	RCT (double-blind, placebo-controlled)	DN	Probiotic (*lactobacillus plantarum A7*)/placebo	0.8 g/kg protein, 2,000 mg sodium, 2,000 mg potassium and 1,500 mg phosphorus	20/20	8	NA	55.25 ± 7.74	26.63 ± 3.29
Borges et al. (7)	Brazil	RCT (double-blind, placebo-controlled)	HD	Probiotic (*Streptococcus thermophilus, Lactobacillus acidophilus, and Bifidobacteria longum*)/placebo	NA	16/17	12	11/10	51.90 ± 9.78	25.25 ± 5.12
Borges et al. (8)	Brazil	RCT (double-blind, placebo-controlled)	HD	Probiotic (*Streptococcus thermophilus, Lactobacillus acidophilus, and Bifidobacteria longum*)/placebo	NA	11/10	12	3/4	54.00 ± 8.16	25.70 ± 4.43
Cosola et al. (20)	Italy	RCT (single-blind, placebo-controlled)	CKD stage 3b-4	Synbiotic (*Lactobacillus Casei, Bifidobacterium Animalis, fructoligosaccharides, and inulin*)/placebo	NA	13/10	8	7/7	51.22 ± 3.65	26.59 ± 1.17
de Andrade et al. (9)	Brazil	RCT (double-blind, placebo-controlled, crossover)	APD	Prebiotic (unripe banana flour-48% resistant starch)/placebo	To maintain a stable dietary pattern and not to take additional microbiological agents during intervention	15/11	12	NA	55.00 ± 12.00	26.7 ± 4.1
Esgalhado et al. (21)	Brazil	RCT (double-blind, placebo-controlled)	HD	Prebiotic (cookies and powder-resistant starch)/placebo	Patients were asked about the amount and type of food eaten at each meal held during the day	15/16	4	7/11	54.71 ± 9.69	26.41 ± 5.07
Eidi et al. (22)	Iran	RCT (triple-blind, placebo-controlled)	HD	Prebiotic (*Lactobacillus Rhamnosus*)/placebo	Not to change their usual dietary intakes	21/21	4	15/17	58.36 ± 14.39	24.46 ± 4.60
Haghighat et al. (23)	Iran	RCT (double-blind, placebo-controlled)	HD	Probiotic (*Lactobacillus acidophilus, Bifidobacterium bifidum, Bifidobacterium lactis, Bifidobacterium longum*)/placebo	To maintain stable dietary intakes, and not to consume any supplements other than the one provided to them by the trial	23/19	12	12/10	45.88 ± 11.04	22.69 ± 4.04
				Synbiotic (*Lactobacillus acidophilus, Bifidobacterium bifidum, Bifidobacterium lactis, Bifidobacterium longum*, fructo-oligosaccharides, galactooligosaccharides, inulin)/placebo		23/19	12	12/10	46.88 ± 10.36	23.64 ± 4.91
Jiang et al. (24)	China	RCT (double-blind, placebo-controlled)	DN	Probiotic (*Bifidobacterium bifidum, Lactobacillus acidophilus, Streptococcus thermophilus*)/placebo	NA	42/34	12	15/12	56.03 ± 8.3	27.03 ± 3.06
Kooshki et al. (25)	Iran	RCT (double-blind, placebo-controlled)	HD	Synbiotic (*Lactobacillus coagulans* and fructo-oligosaccharides)/placebo	Not to change their dietary habits	23/23	8	10/11	62.88 ± 16.52	23.53 ± 4.08
Mafi et al. (26)	Iran	RCT (double-blind, placebo-controlled)	HD	Probiotic (*Lactobacillus acidophilus* strain, *Bifidobacterium bifidum* strain, *Lactobacillus reuteri* strain, and *Lactobacillus fermentum* strain)/placebo	NA	30/30	12	NA	59.9 ± 6.97	25.80 ± 2.81
Miraghajani et al. (27)	Iran	RCT (double-blind, placebo-controlled)	DN	Probiotic (soy milk-*Lactobacillus plantarum* A7)/placebo	Participants received individualized dietary counseling aimed at achieving a daily energy and restricting dietary protein, sodium, and potassium intake.	20/20	8	12/10	55.25 ± 2.37	25.80 ± 2.81
Ramos et al. (28)	Brazil	RCT (double-blind, placebo-controlled)	NDD-CKD	Prebiotic (fructo-oligosaccharides)/placebo	Keep a diet composed by 0.6–0.8 g/kg/day of protein, 30–35 kcal/kg/day of energy, restricted in sodium and controlled in potassium if necessary	23/23	12	13/14	57.50 ± 14.55	26.63 ± 0.71
Simeoni et al. (29)	Italy	RCT (open-label, placebo-controlled)	CKD stage 3a	Probiotic (Lactobacillales and Bifidobacteria)/placebo	Protein dietary intake ranging 0.7–1 g/ kg/day, daily consumption of two pieces of fruit (apple or pear) and 200 g of double-boiled leafy green vegetables	14/14	12	9/6	59.75 ± 5.83	27.60 ± 4.96
Soleimani et al. (30)	Iran	RCT (double-blind, placebo-controlled)	DN + HD	Probiotic (*Lactobacillus acidophilus, Lactobacillus casei* and *Bifidobacterium bifidum*)/placebo	Not to change usual diets and not take any anti-inflammatory and antioxidant medications or supplements during the intervention.	30/30	12	20/20	56.70 ± 16.10	25.85 ± 5.44
Soleimani et al. (31)	Iran	RCT (double-blind, placebo-controlled)	DN + HD	Synbiotic (*Lactobacillus acidophilus, Lactobacillus casei*, and *Bifidobacterium bifidum*, and inulin)/placebo	Not to change usual diets and not take any anti-inflammatory and antioxidant medications or supplements during the intervention	30/30	12	21/21	62.8 ± 13.67	25.70 ± 2.90
Guida et al. (32)	Italy	RCT (double-blind, placebo-controlled)	KTRs	Synbiotic (Lactobacillales, Bifidobacteria, *Streptococcus thermophilus*), prebiotic inulin, and tapioca-resistant starch)/placebo	Mediterranean pattern diet; energy intake higher than 25/kcal/kg/ideal body weight/day, 55% of carbohydrates, total fat not exceeding 30% of calories (fatty acids <10% of calories and dietary cholesterol limited to 300 mg/day); protein intake is restricted to 0.8 g/kg of ideal body weight/day; insoluble and soluble fibers in a ratio of about 3 to 1; water intake ranges between 1.5 and 2.0 L/day	22/12	4	16/12	51.64 ± 9.22	26.65 ± 5.03
Shariaty et al. (33)	Iran	RCT (double-blind, placebo-controlled)	HD	Probiotic (*Lactobacillus acidophilus*, Bifidobacterium and *Streptococcus thermophilus*)/placebo	Both groups received daily folic acid supplements and monthly vitamin B12 supplements	17/17	4	10/10	57.84 ± 11.83	NA
Sirich et al. (34)	The United States	RCT (single-blind, placebo-controlled)	HD	Prebiotic (resistant starch)/placebo	NA	20/20	6	11/13	56.00 ± 13.49	25.34 ± 2.85
Viramontes-Hörner et al. (35)	Mexico	RCT (double-blind, placebo-controlled)	HD	Synbiotic (Lactobacillus acidophilus, Bifidobacterium lactis, Inulin)/placebo	Individualized dietary prescription: energy (30–35 kcal/kg/day), protein intake (1.1–1.2 g/kg/day), as well as potassium, phosphorus, and sodium restriction	22/20	8	16/16	39.84 ± 16.4	29.00 ± 6.44
Mazruei et al. (36)	Iran	RCT (double-blind, placebo-controlled)	DN	Probiotic (Bacillus coagulans)/placebo	NA	30/30	12	NA	61.5 ± 8.81	23.42 ± 5.10
Xie et al. (37)	China	RCT (parallel group, placebo-controlled)	HD	Prebiotic (water soluble fiber, 10 g/day)/placebo	Caloric intake 35 kcal/kg bw, protein intake 1–1.2 g/kg bw, fats <35%, and with sodium and potassium restriction.	41/44	6	24/26	53.39 ± 13.56	30.70 ± 5.10
				Prebiotic (water soluble fiber, 20 g/day)/placebo		39/44	16	18/26	52.44 ± 14.36	22.66 ± 2.10
Rossi et al. (15)	Australia	RCT (double-blind, placebo-controlled, crossover)	CKD stage 4–5	Synbiotic (Lactobacillus, Bifidobacteria, *Streptococcus genera*, and inulin, fructo-oligosaccharides, and galacto-oligosaccharides)/placebo	NA	17/20	16	7/14	68.54 ± 9.87	22.47 ± 1.97
Khosroshahi et al. (38)	Iran	RCT (double-blind, placebo-controlled)	HD	Prebiotic (HAM-RS2)/placebo	NA	22/22	8	12/16	56.00 ± 13.08	26.25 ± 6.01
Khosroshahi et al. (39)	Iran	RCT (double-blind, placebo-controlled)	HD	Prebiotic (HAM-RS2)/placebo	Not to change usual diets	23/21	8	14/15	55.43 ± 11.88	23.52 ± 2.01
Laffin et al. (40)	Canada	RCT (double-blind, placebo-controlled)	HD	Prebiotic (HAM-RS2)/placebo	NA	9/11	8	6/7	55.89 ± 10.25	24.14 ± 1.93
Mirzaeian et al. (41)	Iran	RCT (double-blind placebo-controlled)	HD	Synbiotic (Lactobacillus, Bifidobacteria, *Streptococcus thermophiles*, and fructo-oligosaccharides)/placebo	Not to consume foods such as yogurt, cheese, and kefir, which probably contain probiotic strains.	21/21	8	14/16	64.02 ± 31.5	24.72 ± 4.59
Dehghani et al. (42)	Iran	RCT (double-blind placebo-controlled)	CKD stage 3–4	Synbiotic (Lactobacillus, Bifidobacteria, *Streptococcus thermophiles*, and fructo-oligosaccharides)/placebo	NA	31/35	6	23/27	61.41 ± 7.63	28.53 ± 4.06

*I, intervention; C, control; n, number; BMI, body mass index; RCT, randomized controlled trial; DN, diabetic nephropathy; HD, hemodialysis; CKD, chronic kidney disease; APD, automated peritoneal dialysis; NDD-CKD, non-diabetic, non-dialysis-dependent CKD; KTRs, kidney transplant patients; NA, not available*.

### Risk of Bias Assessment and Network Structures

The risk of bias is summarized in Supplementary Figure S1. Among the 28 trials, 82.1% (23/28) of the RCTs were shown to have a low risk of random sequence generation, while the remaining 5 trials were unclear. The same proportion (82.1%) of low risk was found in the allocation concealment. The risk of performance bias was low in 89.3% (25/28) of trials, except for one with high risk and another two with unclear risk of bias. There were 71.4% (20/28) of RCTs at low risk of blinding for outcome assessment, with 2 at high risk and 6 at unclear risk. Most trials were at low risk of bias for incomplete outcome data, other than the 2 RCTs with high risk. There was no selective reporting in any of the trials. Moreover, 4 trials were identified as having a high risk of bias due to a high rate of loss to follow-up over 20%.

Notably, consistency and inconsistency tests could not be performed because all the outcomes were open-loop network plots. However, the consistency, transitivity, and heterogeneity of all the studies were acceptable for a similar methodology in all the RCTs.

### Treatment Effect on Renal Function

**Figure 2:**
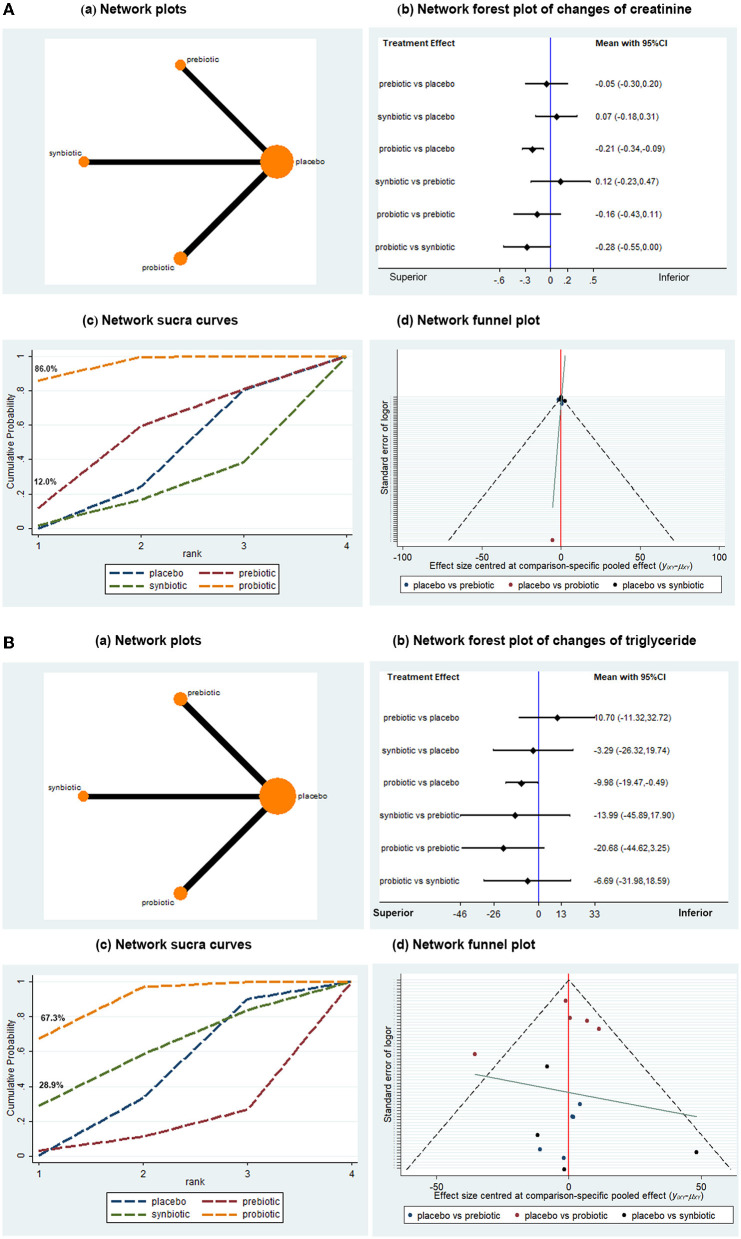
Network meta-analysis results for the effects of probiotic, prebiotic, and synbiotic supplementation on creatinine **(A)** and triglyceride **(B)** levels.

A total of 13 articles with 573 patients provided data on the change in creatinine from baseline. The direct comparison of probiotics, prebiotics, and synbiotics with placebo and indirect treatment comparison among the three microbial supplements are presented in Figure 2A. Probiotics were better than placebo in reducing creatinine (MD −0.21, 95% CI −0.34, −0.09), while prebiotics and synbiotics showed no effects on the reduction of creatinine (MD −0.05, 95% CI −0.30, 0.20; MD 0.07, 95% CI −0.18, 0.31, respectively), suggesting that probiotics were the only microbial supplements that might be renoprotective. In the SUCRA, the probability of probiotics being the best treatment was ~86.0%, where the surface area under the curve of probiotics was larger than the other three, suggesting that it might be the best intervention for the improvement of creatinine. Subgroup analysis was performed according to the intervention period and dialysis status (including hemodialysis and peritoneal dialysis). As shown in Table 2, the effect of probiotics on lowering creatinine was only observed in non-dialysis patients (*n* = 6, MD −0.15, 95% CI −0.20, −0.09) and sufficient intervention duration (≥3 months, *n* = 6, MD −0.29, 95% CI −0.41, −0.17), while no significant difference was found in dialysis patients or the short intervention period (<3 months). A sensitivity analysis that removed an independent article did not affect the overall results, indicating that the conclusion was convincing and reliable. Publication bias was demonstrated to be low by a network funnel plot with perfect symmetry.

**Table 2:** Subgroup analysis of renal function.

**Subgroup by**	**No. of trials**	**No. of patients**	**Probiotics**	**Prebiotics**	**Synbiotics**
**Changes of creatinine**					
Follow-up period					
<3 months	7	288	−0.14 (−1.68, 1.40)	−0.77 (−2.30, 0.75)	0.29 (−0.72, 1.31)
≥3 months	6	285	−0.29 (−0.41, −0.17)^*^	0.00 (−0.19, 0.20)	–
Target population					
Dialysis patients	7	278	−0.31 (−2.98, 2.28)	−0.47 (−2.38, 1.44)	1.02 (−1.15, 3.19)
Non-dialysis patients	6	295	−0.15 (−0.20, −0.09)^*^	0.00 (−0.19, 0.19)	0.04 (−0.17, 0.26)
**Changes of eGFR**					
Follow-up period					
<3 months	4	163	12.70 (6.70, 18.70)^*^^†^	–	1.13 (−1.24, 3.51)
≥3 months	6	307	2.19 (−2.63, 7.02)	0.00 (−8.64, 8.64)	1.00 (−8.95, 10.95)
Target population					
Dialysis patients	2	120	0.49 (−0.27, 1.25)	–	–
Non-dialysis patients	8	350	3.65 (−1.84, 9.13)	0.00 (−8.34, 8.34)	1.82 (−3.52, 7.16)

* *P < 0.05 compared with placebo*,

† *P < 0.05 compared with synbiotics*.

*eGFR, estimated glomerular filtration rate*.

Another indicator of renal function (eGFR) was analyzed in 10 studies of 470 subjects. Compared with placebo, probiotics might have potential advantages in increasing eGFR in patients with CKD with an intervention period of fewer than 3 months (*n* = 4, MD 12.70, 95% CI 6.70, 18.70, Table 2). However, there seemed to be no significant difference in eGFR enhancement among probiotic, prebiotic, and synbiotic supplements in general (Supplementary Figure S2A).

### Treatment Effect on Lipid Profiles

The TG and TC in the lipid profiles were included in the analysis. A total of 14 studies with 732 participants were involved in the assessment of TG change (Figure 2B). Network analysis indicated that probiotics were superior to placebo in terms of lowering TG levels (MD −9.98, 95% CI −19.47, −0.49), while synbiotics and prebiotics were not statistically associated with changes in TG. The probability of probiotics being the best microbial intervention was approximately 67.3%, with the largest surface area. Moderate publication bias was found in the network funnel plot with basic symmetry. Subgroup analysis demonstrated that probiotics might significantly reduce the TG levels of patients with CKD who took probiotics for <3 months or nondialysis patients with CKD (*n* = 8, MD −9.00, 95% CI −14.45, −3.55; *n* = 5, MD −10.70, 95% CI −17.53, −3.87, respectively). However, a significant difference was not found in the patients on dialysis and the trials with a follow-up time over 3 months (Table 3).

**Table 3:** Subgroup analysis of lipid profile.

**Subgroup by**	**No. of trials**	**No. of patients**	**Probiotics**	**Prebiotics**	**Synbiotics**
**Changes of TG**					
Follow-up period					
<3 months	8	418	−10.70 (−17.53, −3.87)^*^	9.22 (−15.32, 33.76)	6.39 (−25.43, 38.20)
≥3 months	6	314	−11.79 (−30.15, 6.57)	15.00 (−36.53, 66.53)	−12.30 (−55.04, 30.44)
Target population					
Dialysis patients	9	524	0.00 (0.00, 1237.4)	1376.79 (0.00, 1.88e+15)	0.00 (0.00, 2.31e+07)
Non-dialysis patients	5	208	−9.00 (−14.45, −3.55)^*^	15.00 (−25.17, 55.17)	44.00 (−11.69, 99.69)
**Changes of TC**					
Follow-up period					
<3 months	8	418	−7.53 (−47.42, 32.36)	−1.01 (−23.44, 21.42)	−11.04 (−37.23, 15.15)
≥3 months	6	314	−5.56 (−11.17, 0.06)	−0.60 (−18.30, 17.10)	−6.60 (−27.53, 14.33)
Target population					
Dialysis patients	9	524	−6.01 (−31.33, 19.31)	−1.18 (−21.09, 18.74)	−13.78 (−37.23, 9.67)
Non-dialysis patients	5	208	−6.65 (−11.59, −1.71)^*^	−0.60 (−18.30, 17.10)	1.90 (−17.42, 21.22)

* *P < 0.05 compared with placebo*.

*TG, triglyceride; TC, total cholesterol*.

The analysis of the change in TC was performed in 14 studies with 732 subjects. There was a tendency for probiotics, prebiotics, and synbiotics to decrease the level of TC, but the statistical significance was not reached (Supplementary Figure S2B). SUCRA showed that probiotics had the best ability to reduce the TC concentration. In the subgroup analysis, the effect of probiotics on lowering TC in non-dialysis patients was statistically superior to placebo (*n* = 5, MD −6.65, 95% CI −11.59, −1.71), while the remaining analysis remained consistent with those described above (Table 3), which indicated whether the individuals had dialysis might be the confounding factor for the efficiency of probiotic on TC decrease.

### Treatment Effect on Inflammatory Biomarkers

**Figure 3:**
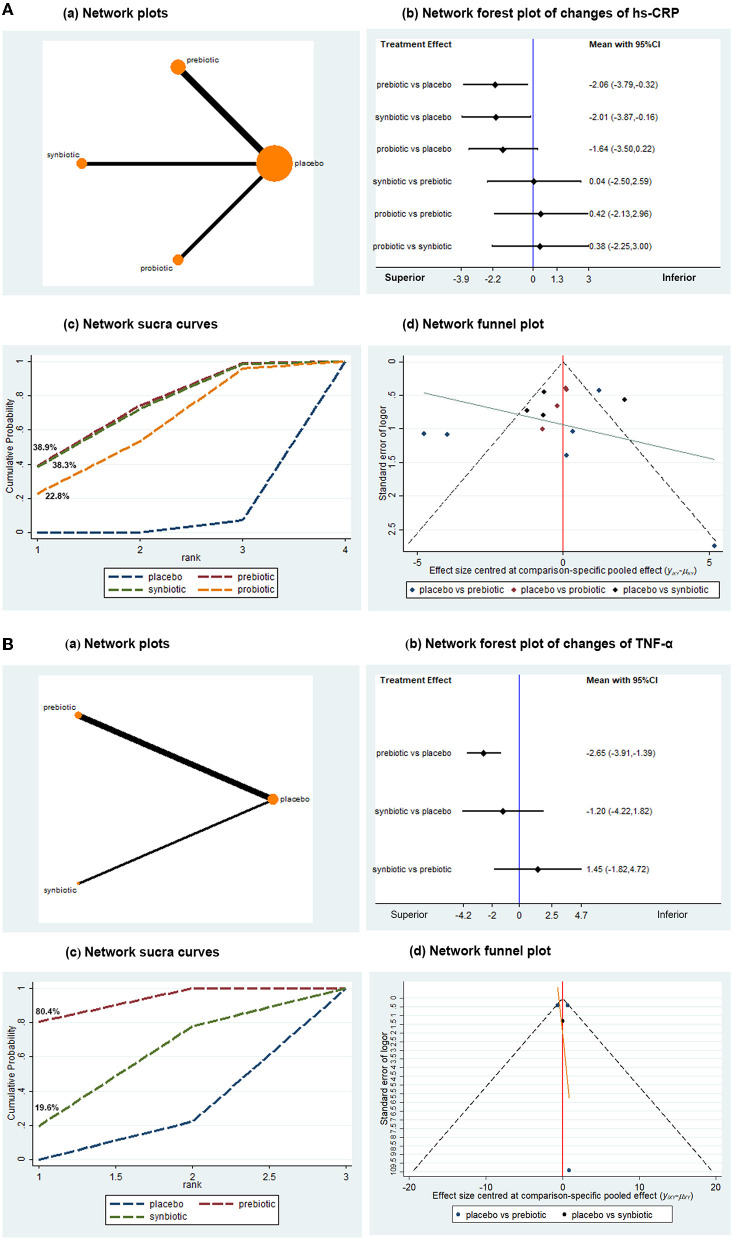
Network meta-analysis results for the effects of probiotic, prebiotic, and synbiotic supplementation on hs-CRP **(A)** and TNF-α **(B)**. hs-CRP, high sensitivity C-reactive protein; TNF-α, tumor necrosis factor-α.

Inflammatory biomarkers, including hs-CRP, IL-6, and TNF-α, were used for statistical analysis. There were 14 studies with 727 patients that evaluated the effects of microbial supplements on hs-CRP. Prebiotics and synbiotics tended to have a better advantage in reducing the levels of hs-CRP (MD −2.06, 95% CI −3.79, −0.32; MD −2.01, 95% CI −3.87, −0.16, respectively), while probiotics showed a tendency to decrease the level of hs-CRP, with no statistically significant difference (MD −1.64, 95% CI −3.50, 0.22) (Figure 3A). SUCRA demonstrated that prebiotics and synbiotics might be the preferred interventions for lowering the concentration of hs-CRP (Figure 3A). A network funnel plot was displayed with moderate publication bias. Subgroup analysis remained consistent in patients who were undergoing dialysis. A marked difference was not observed in predialysis patients, but probiotics showed superiority to placebo in non-dialysis patients (*n* = 2, MD −0.67, 95% CI −1.19, −0.15). When subgrouped by follow-up time, synbiotics and probiotics were associated with the superior effect (*n* = 8, MD −2.74, 95% CI −3.49, −1.98; MD −1.49, 95% CI −1.99, −0.98, respectively) in the long intervention duration (≥3 months), while prebiotics showed no superiority (Table 4). Regarding the follow-up of less than 3 months, no obvious difference was seen among the three groups.

**Table 4:** Subgroup analysis of inflammatory biomarkers.

**Subgroup by**	**No. of trials**	**No. of participants**	**Probiotics**	**Prebiotics**	**Synbiotics**
**Changes of hs-CRP**					
Follow-up period					
<3 months	6	331	–	−2.71 (−6.28, 0.86)	−1.19 (−5.70, 3.32)
≥3 months	8	396	−1.49 (−1.99, −0.98)^*^	−0.14 (−0.92, 0.65)	−2.74 (−3.49, −1.98)^*^
Target population					
Dialysis patients	12	621	−1.62 (−3.75, 0.52)	−2.68 (−4.69, −0.67)^*^	−2.01 (−3.86, −0.17)^*^
Non-dialysis patients	2	106	−0.67 (−1.19, −0.15)^*^	0.00 (−0.58, 0.58)	–
**Changes of TNF-α**					
Follow-up period					
<3 months	3	210	–	−2.65 (−3.93, −1.38)^*^	−1.20 (−4.23, 1.83)
≥3 months	1	16	–	−0.07 (−0.85, 0.71)	–
Target population					
Dialysis patients	4	236	–	−2.65 (−3.91, −1.39)^*^	−1.20 (−4.22, 1.82)
Non-dialysis patients	0	0	0	0	0
**Changes of IL-6**					
Follow-up period					
<3 months	5	261	–	−17.24 (−20.28, −14.20)^*^	0.90 (−0.46, 2.26)
≥3 months	5	189	−7.83 (−21.19, 5.52)	−0.65 (−1.45, 0.15)	−29.93 (−45.72, −14.14)^*^
Target population					
Dialysis patients	9	404	−4.67 (−30.96, 21.62)	−12.87 (−25.20, −0.55)^*^	−11.75 (−30.37, 6.87)
Non-dialysis patients	1	46	–	−0.48 (−1.07, 0.10)	–

* *P < 0.05 compared with placebo*.

*hs-CRP, high sensitivity C-reactive protein; TNF-α, tumor necrosis factor-α; IL-6, interleukin-6*.

In the assessment of TNF-α change from baseline in 4 trials with 236 subjects, prebiotics were associated with a significant reduction in TNF-α (MD −2.65, 95% CI −3.91, −1.39) when compared with placebo (Figure 3B). Data on probiotics were absent. The probability of prebiotics being the best was ~80.4%, with the largest surface area. No evidence of publication bias was found by the network funnel plot. In the subgroup analysis, the results remained consistent in terms of prebiotics lowering TNF-α in the short intervention duration (<3 months) (*n* = 3, MD −2.65, 95% CI −3.93, −1.38). There was only one RCT conducted over 3 months, where statistical analysis could not be performed. There were no data on non-dialysis patients (Table 4).

Ten RCTs with 450 participants were available for the evaluation of the change in IL-6 from baseline. Prebiotic treatment showed a tendency to lower the IL-6 level, with no statistically significant difference (MD −10.24, 95% CI −20.71, 0.24) (Supplementary Figure S3). In the subgroup analysis, prebiotics were shown to have superiority to placebo in patients on dialysis or with a short intervention period (<3 months) (*n* = 5, MD −12.87, 95% CI −25.20, −0.55; MD −17.24, 95% CI −20.28, −14.20, respectively). In the follow-up over 3 months, synbiotics were associated with a significant decrease in IL-6 compared with placebo (*n* = 5, MD −29.93, 95% CI −45.72, −14.14) (Table 4).

### Treatment Effect on Oxidative Stress Indicators

**Figure 4:**
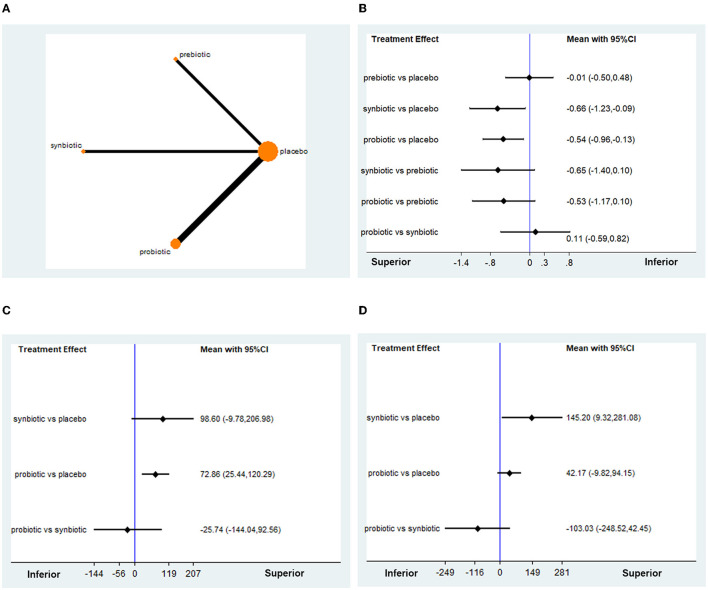
Network meta-analysis results for the effects of probiotic, prebiotic, and synbiotic supplementation on oxidative stress indicators. **(A)** The network structures. **(B)** MDA. **(C)** GSH. **(D)** TAC. MDA, malondialdehyde; GSH, glutathione; TAC, total antioxidant capacity.

The indicators of oxidative stress analyzed in this study included MDA, GSH, and TAC (Figure 4A). Regarding the change in MDA from baseline, 8 RCTs with 494 participants were available for network meta-analysis. The treatment effects of probiotics and synbiotics were superior to those of placebo (MD −0.54, 95% CI −0.96, −0.13; MD −0.66, 95% CI −1.23, −0.09, respectively), while prebiotics showed no significant difference from placebo (MD −0.01, 95% CI −0.50, 0.48) (Figure 4B). SUCRA curves suggested that the probability of synbiotic being the best treatment was approximately 62.3%, followed by probiotics, confirming that synbiotics and probiotics were greater interventions on the decline of MDA than prebiotics (Supplementary Figure S4A). The network funnel plot showed moderate publication bias (Supplementary Figure S4B). Sensitivity analysis did not change the results. The outcomes of the subgroup analysis were similar to those above, especially in patients with CKD on dialysis (probiotics, MD −0.68, 95% CI −0.97, −0.38; synbiotics, MD −0.74, 95% CI −0.96, −0.52, respectively). However, during the follow-up of <3 months, only synbiotics were demonstrated to be superior to placebo in terms of reducing MDA (MD −0.9, 95% CI −1.30, −0.50), whereas only probiotics showed a significant reduction in MDA over 3 months of follow-up (MD −0.73, 95% CI −0.93, −0.53) (Table 5).

**Table 5:** Subgroup analysis of oxidative stress.

**Subgroup by**	**No. of trials**	**No. of patients**	**Probiotics**	**Prebiotics**	**Synbiotics**
**Changes of MDA**					
Follow-up period					
<3 months	4	254	0.01 (−0.06, 0.08)	−0.01 (−0.01, −0.01)	−0.9 (−1.30, −0.50)^*^
≥3 months	4	240	−0.73 (−0.93, −0.53)^*^	–	−0.40 (−0.84, 0.04)
Target population					
Dialysis patients	6	394	−0.74 (−0.96, −0.52)^*^	−0.01(−0.01, −0.01)	−0.68 (−0.97, −0.38)^*^
Non-dialysis patients	2	100	−0.4 (−1.31, 0.52)	–	–
**Changes of GSH**					
Follow-up period					
<3 months	1	40	1.95 (1.20, 2.71)^*^	–	–
≥3 months	4	240	56.52 (1.62, 111.41)^*^	–	98.60 (−7.45, 204.65)
Target population					
Dialysis patients	3	180	73.23 (−9.08, 155.54)	–	98.60 (−22.22, 219.42)
Non-dialysis patients	2	100	1.12 (−0.45, 2.70)	–	–
**Changes of TAC**					
Follow-up period					
<3 months	1	40	0.62 (−0.01, 1.26)	–	–
≥3 months	3	180	0.49 (0.13, 0.86)^*^	–	0.58 (0.06, 1.10)^*^
Target population					
Dialysis patients	3	180	0.49 (0.13, 0.86)^*^	–	0.58 (0.06, 1.10)^*^
Non-dialysis patients	1	40	0.62 (−0.01, 1.26)	–	–

* *P < 0.05 compared with placebo*.

*MDA, malondialdehyde; GSH, glutathione; TAC, total antioxidant capacity*.

A total of 5 articles involving 280 patients investigated the change in GSH. Probiotics were shown to exert a superior effect on improving the level of GSH (MD 72.86, 95% CI 25.44, 120.29), while synbiotics only showed the potential to increase GSH, with no significant difference (Figure 4C). SUCRA revealed a better ability of synbiotics and probiotics to increase GSH (Supplementary Figure S4C). A low publication bias was observed in the network funnel plot (Supplementary Figure S4D). When subgroup analysis was performed based on follow-up time and target population, it was demonstrated that probiotics still had an advantage in elevating GSH level, regardless of short or long follow-up duration (Table 5).

Data on the change in TAC from baseline were available for analysis in four studies with 220 patients. It was found that synbiotics might improve the level of TAC (MD 145.20, 95% CI 9.32, 281.08), while probiotics might not be as effective (MD 42.17, 95% CI −9.28, 94.15, Figure 4D). SUCRA indicated that the probability of synbiotics being the best option was approximately 85.6%, suggesting that the intervention of synbiotics was the most appropriate one concerning improving antioxidant capacity (Supplementary Figure S4E). In the subgroup analysis, an obvious increase in TAC was observed in the probiotic group with patients on dialysis and more than 3 months of follow-up, which signified that the dialysis status and intervention duration might be important confounding factors for the treatment effect of probiotics. The outcomes from a subgroup analysis of synbiotics were stable (Table 5). As shown in Supplementary Figure S4F, the publication bias was moderate, which might be caused by the RCT comparing the treatment effect of probiotics and placebo (31). Sensitivity analysis excluding data from the article by Soleimani showed that probiotics might improve TAC (MD 24.80, 95% CI 4.29, 45.31).

## Discussion

Considering the importance of intestinal microbiota on the progression of CKD and the lack of high-quality evidence that directly compares probiotics, prebiotics, and synbiotics, we performed a network meta-analysis that included 28 RCTs to quantitatively assess the effect of probiotics, prebiotics, and synbiotics on renal function and metabolic profiles in patients with CKD. Overall, probiotics, prebiotics, and synbiotics were determined to be associated with improvements in renal function, lipid profiles, inflammatory biomarkers, and oxidative stress indicators in patients with CKD. More specifically, probiotics were shown to have greater effects in improving renal function (creatinine), lipid profile (TG), and oxidative stress status (MDA, GSH), whereas prebiotics were more advantageous in lowering inflammatory factors (hs-CRP, TNF-α), and synbiotics were represented as a partially synergistic function of probiotics and prebiotics, capable of improving oxidative stress status (MDA and TAC) and inflammatory factors (hs-CRP).

Our study indicated that probiotics could lower serum creatinine and TG levels better than prebiotics and synbiotics, which might delay the progression of CKD. Notably, probiotics were more suitable for non-dialysis patients who would consume them for at least 3 months or more. Previous studies have disputed whether microbial supplements are beneficial to kidney function in patients with CKD. A meta-analysis synthesized 7 RCTs with 456 patients and showed that probiotic intake might significantly decrease serum creatinine, which was consistent with our conclusion (43). However, other published studies found no significant association between probiotic consumption and renal function improvement (11, 44, 45). The reasons for the heterogeneity of the conclusions might be that different meta-analyses included different documents, the design methods were not exactly the same, and the rigorous subgroup analysis was not performed. The articles included in our study were relatively complete. At the same time, we directly and indirectly compared the efficacy of various microbial supplements and conducted subgroup analysis according to the follow-up time and patient characteristics. Therefore, our conclusions might be more reliable. Regarding eGFR, although probiotics showed a tendency to increase its level, there was no statistical significance, which might be attributable to the limited sample size. Subgroup analysis suggested that intervention duration may be a strong confounding factor, and more studies on long-term administration and large sample sizes are needed in the future.

Dyslipidemia was proven to be a pivotal contributing factor for CVD development, which is the main leading cause of morbidity and mortality in patients with CKD (46). Mafi et al. demonstrated that microbial supplements could result in a significant reduction in blood lipids, which was consistent with our findings (26). However, our study also reported that probiotics would be the preferred choice for lowering both cholesterol and TG compared with synbiotics and prebiotics, which might help clinicians to choose microbial supplements. It should be noted that a previous meta-analysis showed contrary results (13). This was mainly because they compared the collective use of probiotics, prebiotics, and synbiotics with a placebo and did not investigate the effects of each microbial supplement. Meanwhile, we included more original studies, and the conclusions might be relatively more stable. Therefore, it was reasonable to conclude that probiotics, among all the microbial supplements, would be the best alternative for improving blood lipid levels in patients with CKD.

Our pooled analysis indicated that the anti-inflammatory effect of prebiotics and synbiotics was marginally better than that of probiotics, especially in reducing hs-CRP and TNF-α. This was mainly caused by the relatively small number of studies on probiotics in this area. One of the critical causative factors that microbial supplements exert anti-inflammatory effects might be attributed to the production of short-chain fatty acids (SCFAs), the major end products of microbial fermentation of prebiotics in the large intestines (47). SCFAs are the major energy source for epithelial cells and can help to stimulate the proliferation and growth of normal epithelial cells, thus stabilizing gut epithelial barrier integrity and reducing the translocation of urotoxins (48). Moreover, SCFAs can reduce inflammatory cytokines such as IL-6 and TNF-α by activating transmembrane G protein-coupled receptors, interfering with the activation of NF-κB induced by lipopolysaccharide (LPS), and/or inhibiting histone acetylation. All these inflammatory markers are reported to be associated with the progression of CVD and ESRD in patients with CKD (49). Therefore, the administration of microbial supplements has greater benefits for patients with CKD.

Although several studies have reported that microbial consumption was able to improve oxidative stress in patients with CKD, our study emphasized the differences in therapeutic effects among these three supplementations, in which probiotics were found to be superior in decreasing serum MDA and increasing GSH, and synbiotics were able to increase TAC levels, while prebiotics did not exert such effects. This discrepancy may provide useful individual guidance for clinical practice. MDA, GSH, and TAC are all well-known biomarkers for oxidative stress in the body. MDA is one of the main products of lipid peroxidation, which could reflect the severity of oxidation stress to a certain extent. GSH, as a major cellular antioxidant metabolite, can eliminate multiple harmful radicals, such as hydrogen peroxides and hydroxyl radicals, and maintain homeostasis of the internal environment from oxidative damage. TAC reflects “the sum of antioxidant activities of the non-specific pool of antioxidants” in the body (50). Oxidative stress was strongly associated with gut microbiota dysbiosis in patients with CKD. Increased intestinal barrier permeability due to high serum urea levels permits the translocation of pathogenic bacteria and their metabolites such as uremic toxins, LPS, and some cytokines, into the circulatory system, eliciting an oxidative stress response as well as systemic inflammation, producing reactive oxygen species (ROS) and releasing proinflammatory cytokines (51). Overproduction of ROS can impose modifications on other oxygen species, DNA, proteins, or lipids due to their highly reactive nature, contributing to a variety of chronic diseases, such as dyslipidemia, CVD, and CKD (52, 53). Meanwhile, it has been reported that oxidative stress and inflammation play an imperative role in the development of renal fibrosis, which can accelerate the loss of kidney function (54, 55). Accordingly, patients with CKD might benefit from probiotics.

Compared with previous meta-analyses, this study has several strengths. To the best of our knowledge, this was the first network meta-analysis to systematically evaluate the effects of probiotic, prebiotic, and synbiotic supplementation on renal function and metabolic profiles in patients with CKD. Based on a comprehensive search of electronic databases, the report included a total of 28 RCTs, which provided a large sample size, making the pooled results convincing. Additionally, we not only concluded that microbial supplements might be beneficial to patients with CKD but also determined which one had the best effect. Moreover, we performed a subgroup analysis of the intervention period and the type of patients, which could indicate the use of different biotic supplements in personalized clinical applications for patients with CKD. It is worth noting that there were some limitations in our study. First, owing to the open-loop of the network plots, consistency and inconsistency tests could not be performed, which made heterogeneity difficult to understand. However, similar methodology in all the RCTs and results of the subgroup analysis indicated that heterogeneity among trials could be accepted. Second, all reviewed probiotic renal outcome studies used creatinine-based measures of renal function. Since probiotics could cause a reduction in creatinine *via* their creatininase activity without a true improvement in intrinsic renal function, probiotic studies are necessary where renal function is more accurately measured using an exogenous maker such as iothalamate or iohexol (56). Third, although our pooled results showed that patients with CKD might benefit from microbial supplementation, the specific mechanism was not clear enough. Consequently, more advanced studies are required in the future.

## Conclusion

The consumption of probiotics, prebiotics, and synbiotics could improve renal function, lipid profiles, inflammatory biomarkers, and oxidative stress indicators in patients with CKD. After thorough consideration, probiotics provide the most comprehensive and beneficial effects for patients with CKD and might be used as the best choice of microecological preparations.

## Data Availability Statement

The original contributions presented in the study are included in the article/Supplementary Material, further inquiries can be directed to the corresponding author/s.

## Author Contributions

WQ, YT, JT, and HZ: study design and data interpretation. HZ and JT: data acquisition and writing. HZ, JT, JD, and JS: statistical analysis. WQ, XZ, and JT: review and editing. All authors contributed to the article and approved the submitted version.

## Funding

This study was partly supported by the National Key R&D Program of China (2020YFC2006500 and 2020YFC2006503) and Grants from the Project of the National Natural Science Foundation of China (No. 81970612).

## Conflict of Interest

The authors declare that the research was conducted in the absence of any commercial or financial relationships that could be construed as a potential conflict of interest.

## Publisher's Note

All claims expressed in this article are solely those of the authors and do not necessarily represent those of their affiliated organizations, or those of the publisher, the editors and the reviewers. Any product that may be evaluated in this article, or claim that may be made by its manufacturer, is not guaranteed or endorsed by the publisher.
