# Supplementary material for: Effectiveness of Microecological Preparations for Improving Renal Function and Metabolic Profiles in Patients With Chronic Kidney Disease
Source: Front Nutr. 2022 Sep 12;9:850014. doi: 10.3389/fnut.2022.850014 (PMC9510395; doi:10.3389/fnut.2022.850014)

## Supplementary Figures

**Supplementary Figure 1.** Risk of bias graph **(A)** and risk of bias summary **(B)** based on the Cochrane Risk of Bias tool for included studies.

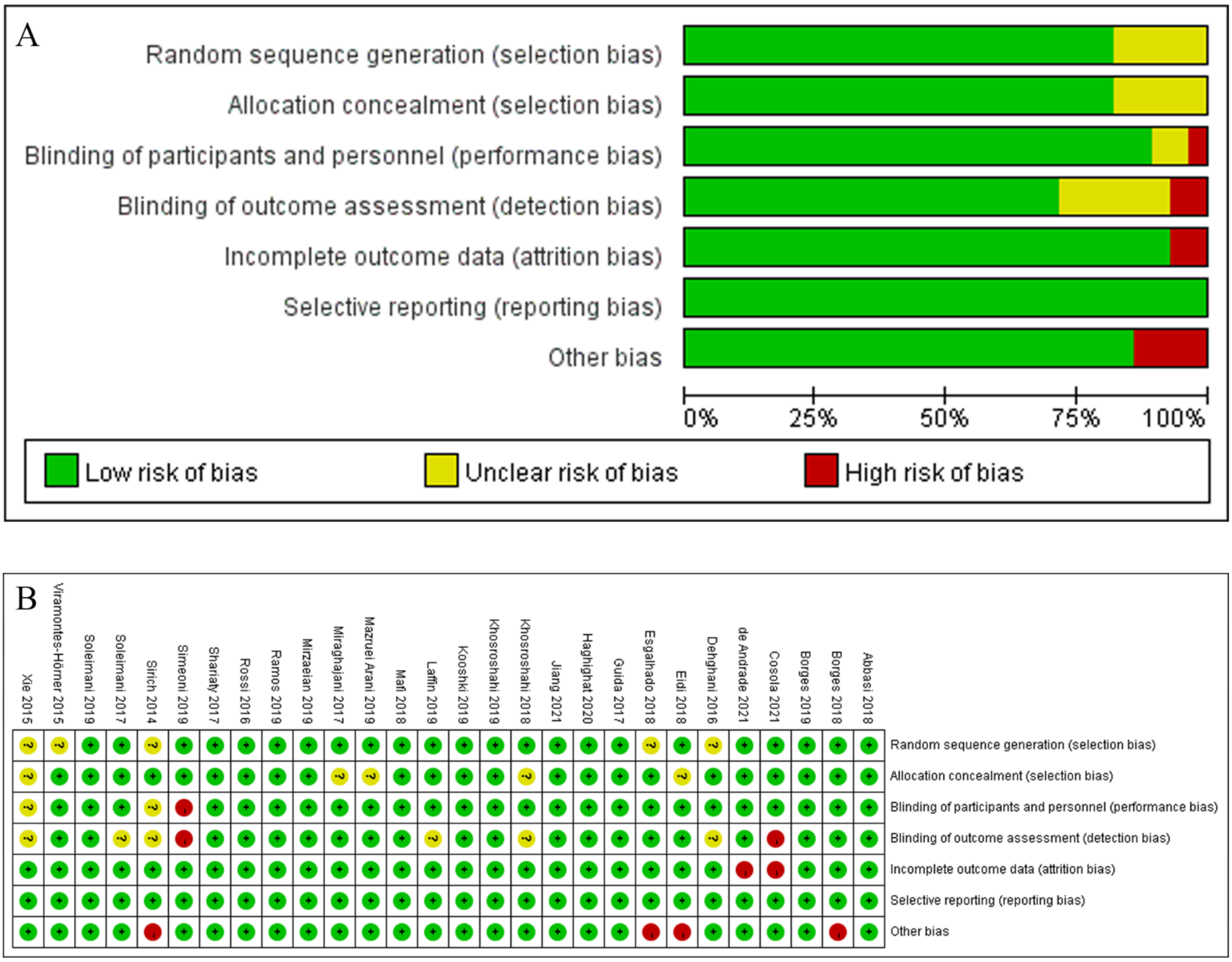

**Supplementary Figure 2.** Network meta-analysis results for effects of probiotic, prebiotic, and synbiotic supplementation on eGFR (A) and TC (B).

Abbreviations. eGFR: estimated glomerular filtration rate. TC: total cholesterol.

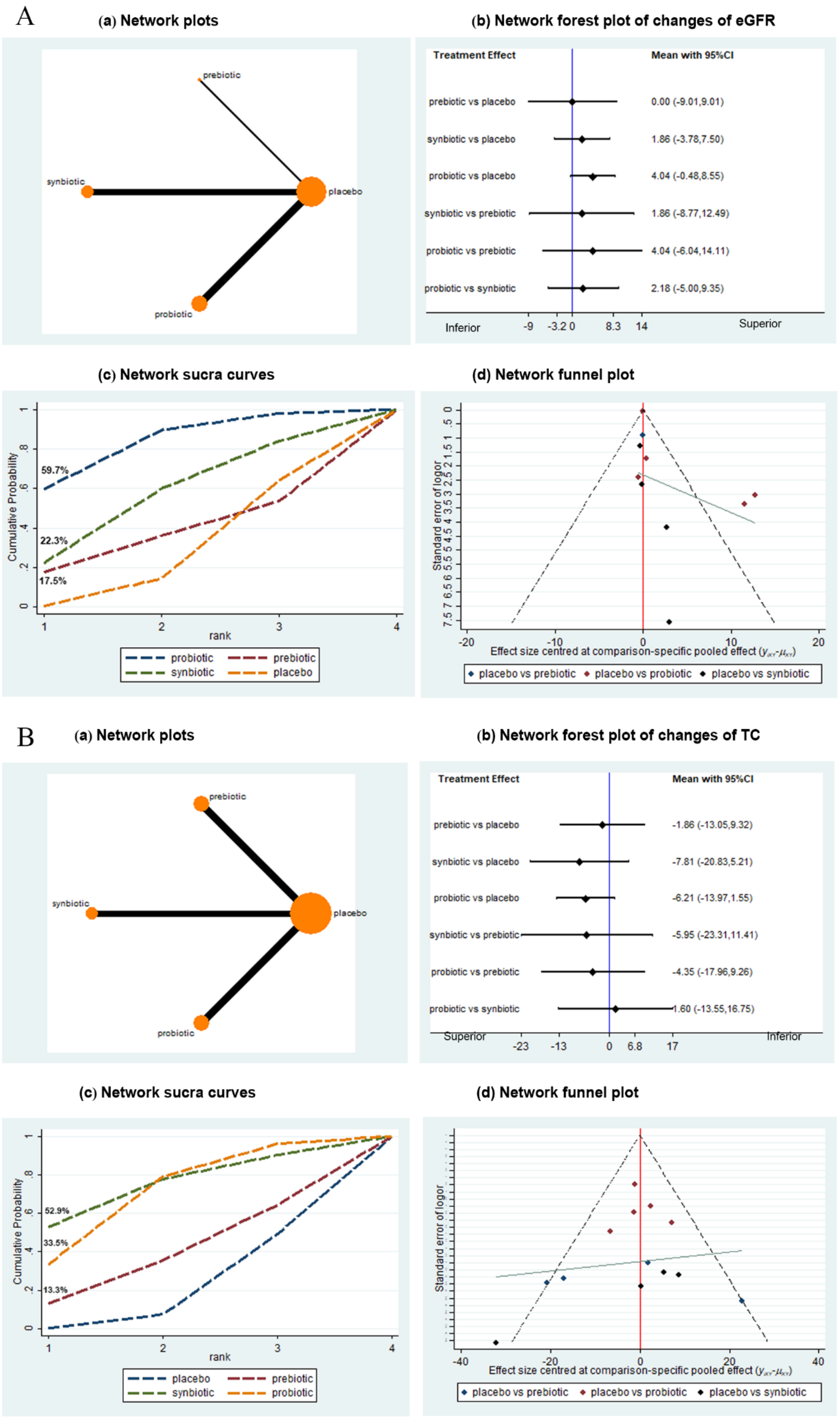

**Supplementary Figure 3.** Network meta-analysis results for effects of probiotic, prebiotic, and synbiotic supplementation on IL-6.

Abbreviations. IL-6: interleukin-6

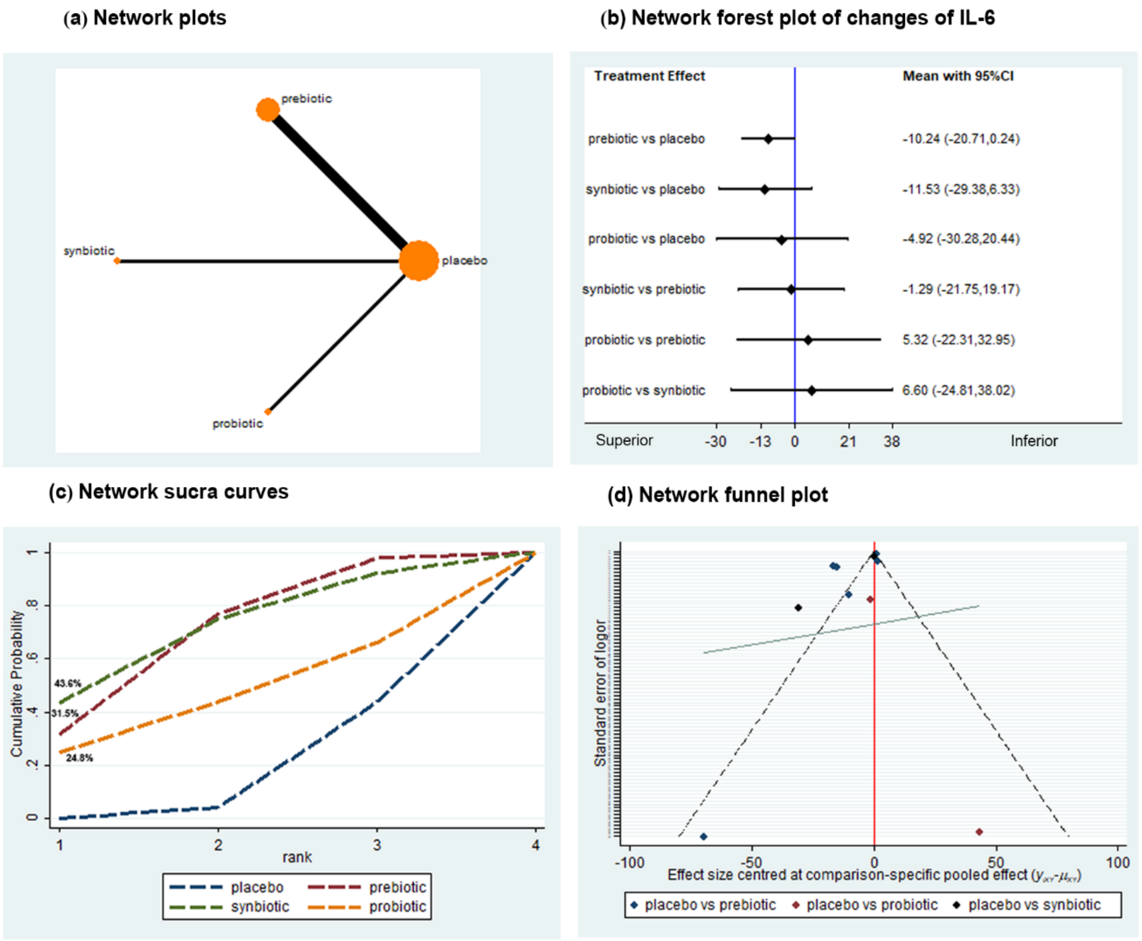

**Supplementary Figure 4.** Network scura curves and funnel plots for effects of probiotic, prebiotic, and synbiotic supplementation on oxidative stress indicators including MDA (a, b); GSH (c, d); TAC (e, f).

Abbreviations. MDA: malondialdehyde. GSH: glutathione. TAC: total antioxidant capacity.

**(a) Network sucra curves of MDA**

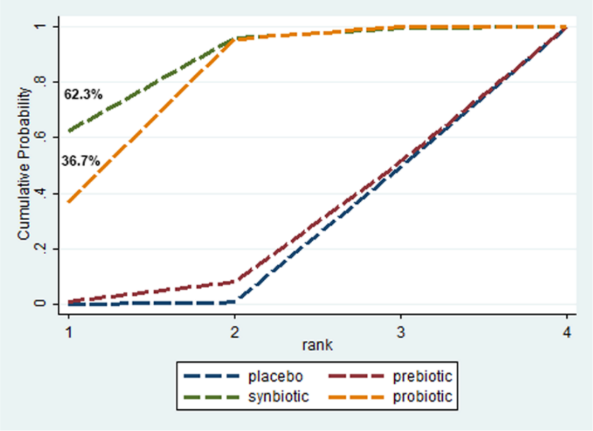


**(b) Network funnel plot of MDA**

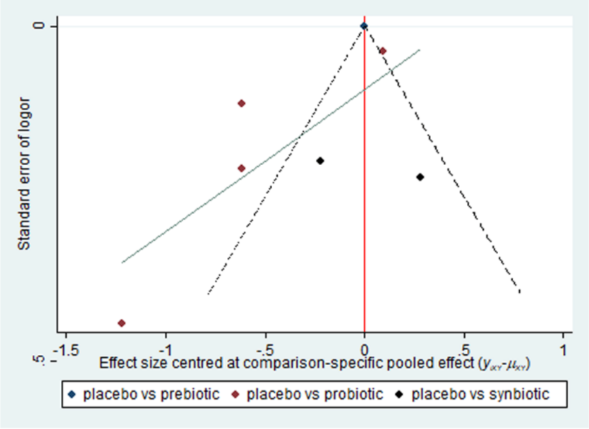


**(c) Network sucra curves of GSH**

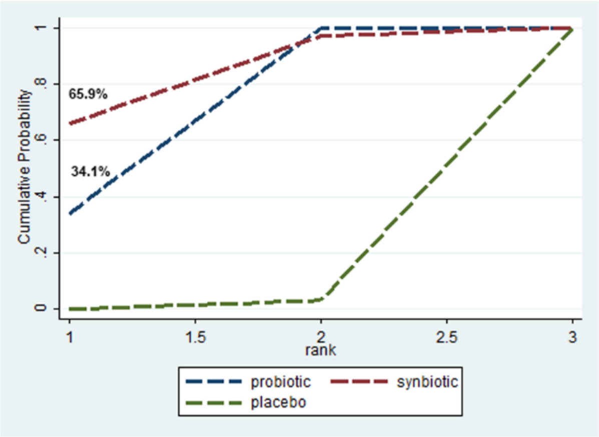


**(d) Network funnel plot of GSH**

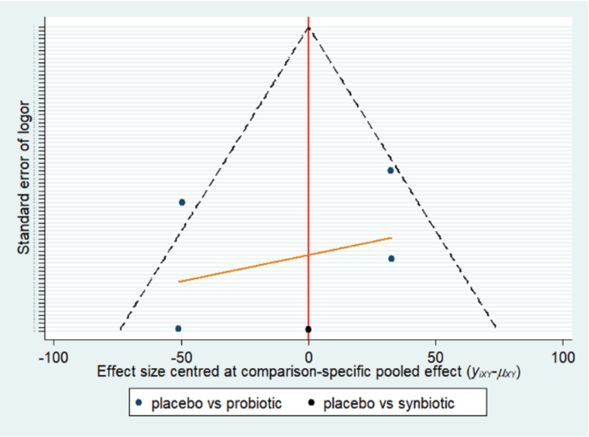


**(e) Network sucra curves of TAC**

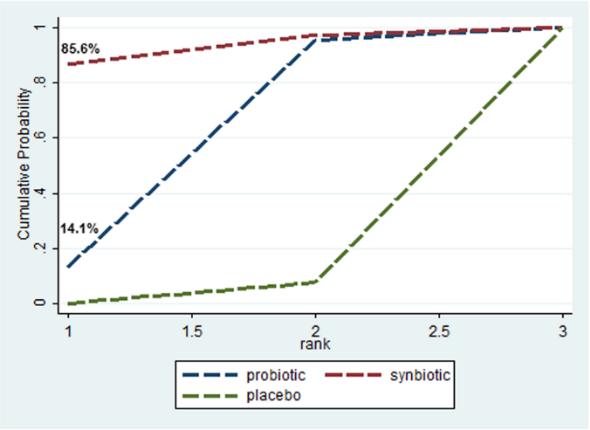


**(f) Network funnel plot of TAC**

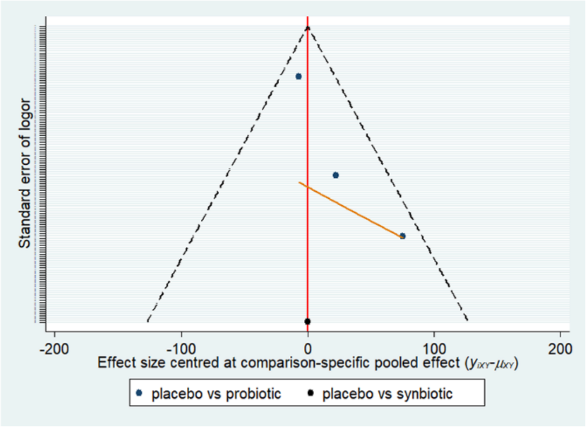

Supplement: Supplementary file 1 [file Data_Sheet_1.PDF]
