# Supplementary material for: Effectiveness of Microecological Preparations for Improving Renal Function and Metabolic Profiles in Patients With Chronic Kidney Disease
Source: Front Nutr. 2022 Sep 12;9:850014. doi: 10.3389/fnut.2022.850014 (PMC9510395; doi:10.3389/fnut.2022.850014)
Supplement: Supplementary file 2 [file Data_Sheet_2.PDF]

Supplementary Table 1

Supplementary Table 1. Search strategy for the systematic review and network meta-analysis

Database	Search terms
OVID MEDLINE	Ovid MEDLINE(R) <1946 to September Week 5 2021><br>1 exp Probiotics/<br>2 exp Prebiotics/<br>3 exp Synbiotics/<br>4 exp Lactobacillus/<br>5 exp Saccharomyces/<br>6 exp Bifidobacterium/<br>7 exp Bacillus/<br>8 exp Yeast, Dried/<br>9 exp Dietary Fiber/<br>10 (probiotic* or prebiotic* or synbiotic* or lactobacillus or dried yeast or bifidobacteri* or Saccharomyce* or bacillus or dietary fiber).tw.<br>11 1 or 2 or 3 or 4 or 5 or 6 or 7 or 8 or 9 or 10<br>12 (chronic kidney disease or chronic kidney failure or chronic renal failure or chronic renal disease).tw.<br>13 (chronic kidney insufficiency or chronic renal insufficiency or chronic renal dysfunction or chronic kidney dysfunction).tw.<br>14 (CKD or CRD or CKF or CRF).tw.<br>15 (end stage renal disease or ESRD or end stage kidney disease or ESKD or ESRF or ESKF or Uremia).tw.<br>16 (glomerulonephritis or dialysis or peritoneal dialysis or hemodialysis or haemodialysis).tw.<br>17 (diabetic nephropathy or renal disease or nephropathy or nephritis).tw.<br>18 exp Renal Insufficiency, Chronic/<br>19 exp Uremia/<br>20 exp Dialysis/<br>21 (CAPD or CCPD or APD).tw.<br>22 (hemofiltration or haemofiltration or hemodiafiltration or haemodiafiltration).tw.<br>23 12 or 13 or 14 or 15 or 16 or 17 or 18 or 19 or 20 or 21 or 22<br>24 exp Randomized Controlled Trial/<br>25 (randomized or randomised or RCT or randomized clinical trials or controlled clinical trial or placebo or randomly or trial).tw.<br>26 24 or 25<br>27 11 and 23 and 26
OVID EMBASE	Embase <1974 to 2021 October 06><br>1 exp probiotic agent/<br>2 exp prebiotic agent/<br>3 exp synbiotic agent/<br>4 exp Lactobacillus/<br>5 exp Saccharomyces/<br>6 exp Bifidobacterium/<br>7 exp Bacillus/<br>8 exp dried yeast/<br>9 exp dietary fiber/<br>10 (probiotic* or prebiotic* or synbiotic* or lactobacillus or dried yeast or bifidobacteri* or Saccharomyce* or bacillus or dietary fiber).tw.<br>11 1 or 2 or 3 or 4 or 5 or 6 or 7 or 8 or 9 or 10<br>12 (chronic kidney disease or chronic kidney failure or chronic renal failure or chronic renal disease).tw.<br>13 (chronic kidney insufficiency or chronic renal insufficiency or chronic renal dysfunction or chronic kidney dysfunction).tw.<br>14 (CKD or CRD or CKF or CRF).tw.<br>15 (end stage renal disease or ESRD or end stage kidney disease or ESKD or ESRF or ESKF).tw.<br>16 (glomerulonephritis or dialysis or peritoneal dialysis or hemodialysis or haemodialysis).tw.<br>17 (diabetic nephropathy or renal disease or nephropathy or nephritis).tw.

	18 exp chronic kidney failure/<br>19 exp uremia/<br>20 exp dialysis/<br>21 (CAPD or CCPD or APD).tw.<br>22 (hemofiltration or haemofiltration or hemodiafiltration or haemodiafiltration).tw.<br>23 12 or 13 or 14 or 15 or 16 or 17 or 18 or 19 or 20 or 21 or 22<br>24 exp randomized controlled trial/<br>25 (randomized or randomised or RCT or randomized clinical trials or controlled clinical trial or placebo or randomly or trial).tw.<br>26 24 or 25<br>27 11 and 23 and 26
Pubmed	((((((probiotic[MeSH Terms]) OR (prebiotic[MeSH Terms])) OR (synbiotic[MeSH Terms])) OR ((Lactobacillus OR Saccharomyces OR Bifidobacterium OR Bacillus OR dried yeast OR dietary fiber))) OR ((probiotic* or prebiotic* or synbiotic* or lactobacillus or dried yeast or bifidobacteri* or Saccharomyce* or bacillus or dietary fiber))) AND (((((((((chronic kidney failure[MeSH Terms]) OR (uremia[MeSH Terms])) OR (dialysis[MeSH Terms])) OR ((chronic kidney disease or chronic kidney failure or chronic renal failure or chronic renal disease))) OR ((chronic kidney insufficiency or chronic renal insufficiency or chronic renal dysfunction or chronic kidney dysfunction))) OR ((CKD or CRD or CKF or CRF))) OR ((end stage renal disease or ESRD or end stage kidney disease or ESKD or ESRF or ESKF))) OR ((glomerulonephritis or dialysis or peritoneal dialysis or hemodialysis or haemodialysis))) OR ((((diabetic nephropathy or renal disease or nephropathy or nephritis)) OR ((CAPD or CCPD or APD))) OR ((hemofiltration or haemofiltration or hemodiafiltration or haemodiafiltration))))) AND ((randomized controlled trial[MeSH Terms]) OR ((randomized or randomised or RCT or randomized clinical trials or controlled clinical trial or placebo or randomly or trial)))
SCIE	#1 TS=(probiotic)<br>#2 TS=(prebiotic)<br>#3 TS=(synbiotic)<br>#4 TS=((Lactobacillus OR Saccharomyces OR Bifidobacterium OR Bacillus OR dried yeast OR dietary fiber))<br>#5 TS=((probiotic* or prebiotic* or synbiotic* or lactobacillus or dried yeast or bifidobacteri* or Saccharomyce* or bacillus or dietary fiber))<br>#6 ((((#1) OR #2) OR #3) OR #4) OR #5<br>#7 TS=((chronic kidney disease or chronic kidney failure or chronic renal failure or chronic renal disease))<br>#8 TS=((chronic kidney insufficiency or chronic renal insufficiency or chronic renal dysfunction or chronic kidney dysfunction))<br>#9 TS=((CKD or CRD or CKF or CRF))<br>#10 TS=((end stage renal disease or ESRD or end stage kidney disease or ESKD or ESRF or ESKF))<br>#11 TS=((glomerulonephritis or dialysis or peritoneal dialysis or hemodialysis or haemodialysis))<br>#12 TS=((diabetic nephropathy or renal disease or nephropathy or nephritis))<br>#13 TS=(Renal Insufficiency, Chronic)<br>#14 TS=(Uremia)<br>#15 TS=(Dialysis)<br>#16 TS=((CAPD or CCPD or APD))<br>#17 TS=((hemofiltration or haemofiltration or hemodiafiltration or haemodiafiltration))<br>#18 ((((((((((#7) OR #8) OR #9) OR #10) OR #11) OR #12) OR #13) OR #14) OR #15) OR #16) OR #17<br>#19 TS=(Randomized Controlled Trial)<br>#20 TS=((randomized or randomised or RCT or randomized clinical trials or controlled clinical trial or placebo or randomly or trial))<br>#21 (#19) OR #20<br>#22 ((#6) AND #18) AND #21
Cochrane	#1 ((“probiotics” OR “prebiotics” OR “synbiotics” OR “Lactobacillus” OR “Yeast, Dried”)):ti,ab,kw<br>#2 MeSH descriptor: [Probiotics] explode all trees<br>#3 MeSH descriptor: [Prebiotics] explode all trees<br>#4 MeSH descriptor: [Synbiotics] explode all trees<br>#5 MeSH descriptor: [Lactobacillus] explode all trees

	#6 MeSH descriptor: [Yeast, Dried] explode all trees<br>#7 #1 OR #2 OR #3 OR #4 OR #5 OR #6<br>#8 ((“chronic kidney disease” OR “chronic kidney failure” OR “chronic renal failure” OR “chronic renal disease” OR “chronic kidney insufficiency” OR “chronic renal insufficiency” OR “chronic renal dysfunction” OR “chronic kidney dysfunction” OR “CKD” OR “CRD” OR “CKF” OR “CRF” OR “end stage renal disease” OR “ESRD” OR “end stage kidney disease” OR “ESKD” OR “ESRF” OR “ ESKF” OR “Uremia” OR “glomerulonephritis” OR “dialysis” OR “peritoneal dialysis” “hemodialysis” OR “haemodialysis” OR “diabetic nephropathy” OR “renal disease” OR “nephropathy” OR “nephritis” OR “CAPD” OR “CCPD” OR “APD” OR “hemofiltration” OR “haemofiltration” OR “hemodiafiltration” OR “haemodiafiltration”)):ti,ab,kw<br>#9 MeSH descriptor: [Renal Insufficiency, Chronic] explode all trees<br>#10 #8 OR #9<br>#11 ((“randomized” OR “randomised “ OR “RCT” OR “randomized clinical trials” OR “controlled clinical trial” OR “placebo” OR “randomly” OR “trial”)):ti,ab,kw<br>#12 MeSH descriptor: [Randomized Controlled Trial] explode all trees<br>#13 #11 OR #12<br>#14 #7 AND #10 AND #13

Supplementary Table 2

Supplementary Table 2. Disposition after full text review

Author	Journal	Decision
Abbasi, B. 2018	Progress in Nutrition	Included
Moravejolahkami 2021	Journal of Diabetes and Metabolic Disorders	Excluded: non RCT
Borges, N. A 2018	Journal of Renal Nutrition	Included
Borges, N. A 2019	Journal of Renal Nutrition	Included
Cosola, C. 2021	Toxins	Included
De Andrade, L. S. 2021	Nutrients	Included
Esgalhado, M. 2018	Food Function.	Included
Eidi, F. 2018	Clinical Nutrition ESPEN	Included
Haghighat, N. 2019	Probiotics and Antimicrobial Proteins	Excluded: irrelevant outcomes
Haghighat, N. 2020	Probiotics and Antimicrobial Proteins	Included
Haghighat, N. 2021	Nutritional Neuroscience	Excluded: irrelevant outcomes
Jiang, H. 2021	Journal of Clinical Laboratory Analysis	Included
Kooshki, A. 2019	Hemodialysis International	Included
Liu, H. 2020	European Journal of Nutrition	Included
Mafi, A. 2018	Food & function	Included
Jia, L. 2018	Kidney and Blood Pressure Research	Excluded: non RCT
Meijers, B. K. 2010	Nephrol Dial Transplant	Excluded: non RCT
Miraghajani. 2017	Journal of Renal Nutrition	Included
Miraghajani, M. 2019	Probiotics and Antimicrobial Proteins	Excluded: irrelevant outcomes
McFarlane 2019	J Ren Nutr	Excluded: non RCT
Wang, H. 2021	MATERIALS EXPRESS	Excluded: non RCT
Pei, M. 2018	BMJ	Excluded: non RCT
Ramos, C. I. 2019	Nephrology, dialysis, transplantation	Included
Tao, S. 2019	Nephrology	Excluded: non RCT
Simeoni, M. 2019	European Journal of Nutrition	Included
Soleimani 2017	Kidney international	Included
Soleimani 2019	Probiotics and antimicrobial proteins	Included
Guida, B. 2017	Journal of the American College of Nutrition	Included

Shariaty, Z. 2017	Journal of research in medical sciences	Included
Sirich, T. L. 2014	Clinical journal of the American Society of Nephrology	Included
Viramontes-Hörner, D. 2015	Journal of renal nutrition	Included
Mazruei Arani, N. 2019	Probiotics and antimicrobial proteins	Included
Xie, L.-M. 2015	International journal of clinical and experimental medicine	Included
Rossi, M. 2016	Clinical journal of the American Society of Nephrology : CJASN	Included
De Paiva, B. R.2020	International urology and nephrology	Excluded: irrelevant outcomes
Khosroshahi 2018	International Symposium on Home Hemodialysis	Included
Poesen 2016	PloS one	Excluded: data missing
Khosroshahi 2019	Nutrition & metabolism	Included
Laffin 2019	International Symposium on Home Hemodialysis	Included
Mirzaeian 2019	Nutrition	Included
Dehghani 2016	Iranian journal of kidney diseases	Included
Ranganathan 2009	Current medical research and opinion	Excluded: data missing
Lopes 2018	Food research international	Excluded: irrelevant outcomes
Lopes 2019	Food research international	Excluded: irrelevant outcomes
Thongprayoon. 2018	Journal of Nephropathology	Excluded: non RCT
Khor 2018	Nutrients	Excluded: non RCT
Pisano 2018	Nutrients	Excluded: non RCT
Liu 2020	Eur J Nutr	Excluded: irrelevant outcomes

Supplementary Table 3

Supplementary Table 3. Characteristics of included studies.

1. Abbasi, B. 2018

Methods	The first author: B. Abbasi 2018<br>Study design: RCT (double-blind)<br>Study duration: 8 weeks
Subjects	Country/Region: Iran<br>Population: diabetic nephropathy<br>Sample size: probiotic soy milk (soy milk containing *lactobacillus plantarum A7*) (20), conventional soy milk (20)<br>Gender (Male):<br>Mean age (± SD): probiotic soy milk (soy milk containing *lactobacillus plantarum A7*) (56.9±8.1), conventional soy milk (53.6±7.19)<br>Inclusive criteria: 25 years of age or older, proven type II diabetes for more than a year with fasting blood glucose higher than 126 mg/dl, 2h postprandial blood glucose higher than 200 mg/dl, microalbuminuria and GFR higher than 60 ml/min.<br>exclusive criteria: Subjects with prior history of inflammatory bowel disease (IBD), infection, liver disease, rheumatoid arthritis, smoking, alcoholism, recent antibiotic therapy, and consuming multivitamin and mineral or omega-3 supplements 1 months prior to beginning of the intervention.
Interventions	Treatment group: probiotic soy milk (soy milk containing *lactobacillus plantarum A7*)<br>Control group: conventional soy milk
Outcomes	Outcomes extracted for meta-analysis:<br>HDL-C, LDL-C, T-Chol, non HDL-C, TG, Serum creatinine, Serum phosphorus, Serum genistein, eGFR

2. Borges, N. A 2018

Methods	The first author: Borges, N. A 2018<br>Study design: RCT (double-blind, placebo-controlled)<br>Study duration: 3 months
Subjects	Country/Region: Brazil<br>Population: hemodialysis (HD) patients<br>Sample size: probiotic (*Streptococcus thermophilus, Lactobacillus acidophilus e Bifidobacterialongum*, 90 billion colony-forming units per day) (16), placebo (17)<br>Gender (Male): probiotic (11), placebo (10)<br>Mean age (± SD): probiotic (53.6±11.0), placebo (50.3±8.5)<br>Inclusive criteria: Patients aged 18 years, undergoing HD for at least 6 months<br>exclusive criteria: Patients with inflammatory diseases, cancer, AIDS, autoimmune disease, smokers, use of a central catheter for hemodialysis access, amputated limbs, pregnancy, and patients who had used catabolic drugs, antioxidant vitamin supplements pre, pro, and symbiotic and antibiotics in the last 3 months before starting this study.
Interventions	Treatment group: probiotic (*Streptococcus thermophilus, Lactobacillus acidophilus e Bifidobacterialongum*, 90 billion colony-forming units per day)<br>Control group: placebo
Outcomes	Outcomes extracted for meta-analysis:<br>inflammatory markers (C-reactive protein and interleukin-6), Uremic toxins plasma levels (indoxyl sulfate, p-cresyl sulfate, and indole-3-acetic acid, trimethylamine-N-oxide, choline, betaine), serum urea, creatinine, hemoglobin, potassium, albumin, globulin, fecal pH

3. Borges, N. A 2019

Methods	The first author: Borges, N. A 2018<br>Study design: RCT (double-blind, placebo-controlled)<br>Study duration: 3 months
Subjects	Country/Region: Brazil<br>Population: hemodialysis (HD) patients<br>Sample size: probiotic (*Streptococcus thermophilus, Lactobacillus acidophilus e Bifidobacterialongum*, 90 billion colony-forming units per day) (11), placebo (10)<br>Gender (Male): probiotic (3), placebo (4)<br>Mean age (± SD): probiotic (54±9.8), placebo (54±6.43)<br>Inclusive criteria: Patients aged 18 years, undergoing HD for at least 6 months<br>exclusive criteria: Patients with inflammatory diseases, cancer, AIDS, autoimmune disease, smokers, use

	of a central catheter for hemodialysis access, amputated limbs, pregnancy, and patients who had used catabolic drugs, antioxidant vitamin supplements pre, pro, and symbiotic and antibiotics in the last 3 months before starting this study.
Interventions	Treatment group: probiotic (*Streptococcus thermophilus, Lactobacillus acidophilus e Bifidobacterialongum*, 90 billion colony-forming units per day)<br>Control group: placebo
Outcomes	Outcomes extracted for meta-analysis:<br>C-reactive protein, trimethylamine-N-oxide, choline, betaine

4. Cosola, C. 2021

Methods	The first author: Cosola, C. 2021<br>Study design: RCT (single-blind, placebo-controlled)<br>Study duration: 2 months
Subjects	Country/Region: Italy<br>Population: stage IIIb-IV CKD patients<br>Sample size: synbiotic (13 CKD), placebo (10 CKD)<br>Gender (Male): synbiotic (7), placebo (7)<br>Mean age (± SD): synbiotic (51 ± 4.3), placebo (51.5 ± 2.8)<br>Inclusive criteria: stage IIIb-IV CKD patients<br>exclusive criteria: declined to participate
Interventions	Treatment group: synbiotic formulation NATUREN G<br>Control group: placebo
Outcomes	Outcomes extracted for meta-analysis:<br>indoxyl sulfate (IS), p-cresyl sulfate (PCS), trimethylamine-N-oxide (TMAO) and indoleacetic acid (IAA), Serum creatinine, Azotemia, Serum sodium etc.

5. de Andrade, L. S. 2021

Methods	The first author: de Andrade, L. S. 2021<br>Study design: RCT (double-blind, placebo-controlled, crossover)<br>Study duration: 3 months
Subjects	Country/Region: Brazil<br>Population: Patients undergoing automated peritoneal dialysis (APD)<br>Sample size: prebiotic + placebo (26)<br>Gender (Male): prebiotic + placebo (14)<br>Mean age (± SD): prebiotic + placebo (55 ± 12)<br>Inclusive criteria: Patients undergoing automated peritoneal dialysis (APD), aged 18–80 years, with a dialysis vintage of at least three months and adherent to dialysis treatment.<br>exclusive criteria: the use of prebiotics, probiotics, synbiotics, and antibiotics one month before the beginning of the study, the presence of inflammatory bowel diseases, stomach or bowel resection, liver cirrhosis, cancer, human immunodeficiency virus, peritonitis in the last month, pregnancy, and breastfeeding.
Interventions	Treatment group: unripe banana flour (UBF—48% resistant starch, a prebiotic)<br>Control group: placebo
Outcomes	Outcomes extracted for meta-analysis:<br>The primary outcomes: serum levels of IS, pCS, and IAA.<br>The secondary outcomes: 24 h urine excretion and dialysis removal of IS, pCS, and IAA, serum inflammatory markers (high-sensitivity C-reactive protein (hsCRP), interleukin-6 (IL-6), interleukin-10 (IL-10), and tumor necrosis factor-α (TNF-α)), serum lipopolysaccharide (LPS), dietary intake, and gastrointestinal symptoms.

6. Esgalhado, M. 2018

Methods	The first author: Esgalhado, M. 2018<br>Study design: RCT (double-blind, placebo-controlled)<br>Study duration: 1 month
Subjects	Country/Region: Brazil<br>Population: hemodialysis patients<br>Sample size: resistant starch (15) + resistant starch (16)<br>Gender (Male): Resistant starch (7) + placebo (11)<br>Mean age (± SD): Resistant starch (56.0 ± 7.5) + placebo (53.5 ± 11.5)<br>Inclusive criteria: males and females older than 18 years who received dialysis treatment for at least 6

	months before the study and who had arteriovenous fistula for vascular access in the upper limb.<br>Exclusive criteria: Patients with autoimmune, infectious or neoplastic diseases, or HIV; patients who take catabolic drugs, antioxidant or vitamin supplements, prebiotics, probiotics, symbiotics, or antibiotics in the three months prior to the start of this study. Patients who were pregnant, and regularly practiced physical exercise were also excluded.
Interventions	Treatment group: Resistant starch (a prebiotic)<br>Control group: placebo
Outcomes	Outcomes extracted for meta-analysis:<br>Urea pre-dialysis, Urea post-dialysis, Creatinine, Phosphorus, Potassium, Hematocrit, Hemoglobin, Albumin, Protein carbonyl, TBARS (thiobarbituric acid reactive substances); IL-6; hs-CRP（high-sensitive C-reactive protein）, p-Cresyl sulfate, Indoxyl sulfate

7. Eidi, F. 2018

Methods	The first author: Eidi, F. 2018<br>Study design: RCT (triple-blind, placebo-controlled)<br>Study duration: 1 month
Subjects	Country/Region: Iran<br>Population: hemodialysis patients<br>Sample size: Lactobacillus Rhamnosus (21) + resistant starch (21)<br>Gender (Male): Resistant starch (15) + placebo (17)<br>Mean age (± SD): Resistant starch (57.05 ± 13.95) + placebo (59.67 ± 15.04)<br>Inclusive criteria: Age more than 20 years, acceptable performance of alimentary system, ability to drink at least 200 ml of water per day, no history of being allergic to fungi and yeast, absence of acute medical illness, life expectancy more than 3 months, accept and sign the consent.<br>Exclusive criteria: Patient's unwillingness to participate in the study History of smoking, peritoneal dialysis (PD) or previous kidney transplantation, lactation or pregnancy, drug history including antibiotics, prebiotics, probiotics, herbal drugs, psychedelic drugs, flavors.
Interventions	Treatment group: Lactobacillus Rhamnosus<br>Control group: placebo
Outcomes	Outcomes extracted for meta-analysis:<br>Urea, Creatinine, Leukocyte, Platelet counts, Liver Function Tests (LFT), Albumin, serum electrolytes including of Na, K, Ca and P concentrations, serum Uremic toxins (Phenol, P-cresol)

8. Haghighat, N. 2020

Methods	The first author: Haghighat, N. 2020<br>Study design: RCT (double-blind, placebo-controlled)<br>Study duration: 3 months
Subjects	Country/Region: Iran<br>Population: hemodialysis patients<br>Sample size: Probiotic (23) + symbiotic (23) +placebo (19)<br>Gender (Male): Probiotic (12) + symbiotic (12) +placebo (10)<br>Mean age (± SD): Probiotic (46.21±11.49) + symbiotic (48.04±10.11) +placebo (45.47±10.76)<br>Inclusive criteria: patients undergoing hemodialysis (HD) at least 3 months before the study started, have the arteriovenous fistula and aged 30 to 65 years.<br>Exclusive criteria: patients with previous kidney transplant or likely to receive a transplant, the presence of inflammatory and infectious diseases, malignancy, chronic liver disease, use of a central catheter for hemodialysis access, amputated limbs, pregnancy, and patients using steroidal and/or nonsteroidal anti-inflammatory drugs and antibiotics, antioxidant, and/or anti-inflammatory supplements (e.g., vitamin E, vitamin C, and omega-3 fatty acids), pre-, pro-, and synbiotic and other forms of probiotics (including probiotic yogurt, kefir, and other fermented foods) within 3 months of study commencement.
Interventions	Treatment group: Probiotic or symbiotic<br>Control group: placebo
Outcomes	Outcomes extracted for meta-analysis:<br>Serum concentrations of creatinine and urea, hs-CRP, IL-6, Endotoxin

9. Jiang, H. 2021

Methods	The first author: Jiang, H. 2021<br>Study design: RCT (double-blind, placebo-controlled)<br>Study duration: 3 months

Subjects	Country/Region: China<br>Population: patients with diabetic nephropathy<br>Sample size: Probiotic (42) +placebo (34)<br>Gender (Male): Probiotic (15) + placebo (12)<br>Mean age (± SD): Probiotic (55.96 ± 8.45) + placebo (56.12 ± 8.23)<br>Inclusive criteria: (1) patient age was ≥18 years and ≤75 years; (2) patients were diagnosed with diabetic nephropathy according to world health organization's standard of type 2 diabetes mellitus21 and did not intake any antidiabetic drugs within 3 months before the study; (3) the patients' glycosylated hemoglobin (HbA1c) levels was between 7% and 10%; and (4) the microalbuminuria/creatinine (mAlb/Cr) level was ≥30 mg/g per 24 h.<br>Exclusive criteria: (1) patients with type 1 diabetes mellitus; (2) patients with hypoglycemic coma, diabetic ketoacidosis, hyperosmotic nonketotic coma or diabetes mellitus acute complications; (3) fasting blood glucose was >13.3 mmol/L; (4) total bilirubin was >2.5 times of normal value; (5) serum creatinine was >133 μmol/L in male patients, and >124 μmol/L in female patients; (6) patients with history of hypertension, drug abuse, alcohol dependence or drug allergy; (7) patients' intake of angiotensin-converting enzyme inhibitors or angiotensin receptor blockers within 3 months; and (8) patients' intake probiotic and/or synbiotic supplements within 3 months before this study.
Interventions	Treatment group: Probiotic (*Bifidobacterium bifidum* 1.2 × 109 CFU, *Lactobacillus acidophilus* 4.2 × 109 CFU, *Streptococcus thermophilus* 4.3 × 109 CFU)<br>Control group: placebo
Outcomes	Outcomes extracted for meta-analysis:<br>Fasting blood glucose, 2 h postprandial blood glucose level, glycosylated hemoglobin (HbA1c) and serum creatinine, Fasting insulin levels, Microalbuminuria/creatinine, Estimated glomerular filtration rate,

10. Kooshki, A. 2019

Methods	The first author: Kooshki, A. 2019<br>Study design: RCT (double-blind, placebo-controlled)<br>Study duration: 2 months
Subjects	Country/Region: Iran<br>Population: hemodialysis patients<br>Sample size: Synbiotic (23) +placebo (23)<br>Gender (Male): Synbiotic (10) + placebo (11)<br>Mean age (± SD): Synbiotic (62.92±16.80)+placebo (62.83±16.62)<br>Inclusive criteria: Hemodialysis patients who were free of overt inflammatory and infectious diseases, including hepatitis.<br>Exclusive criteria: Patients who were receiving Nigella sativa oil, omega-3 fatty acid, L-carnitine, vitamin E and/or C supplements, or steroidal and/or nonsteroidal anti-inflammatory drugs.
Interventions	Treatment group: synbiotic supplement *Lactobacillus coagulans* and *fructo-oligosaccharides*<br>Control group: placebo
Outcomes	Outcomes extracted for meta-analysis:<br>serum C-reactive protein (hs-CRP) and malondialdehyde (MDA), total cholesterol, TGs, low density lipoprotein cholesterol (LDL-c), HDL-C

11. Mafi, A. 2018

Methods	The first author: Mafi, A. 2018<br>Study design: RCT (double-blind, placebo-controlled)<br>Study duration: 3 months
Subjects	Country/Region: Iran<br>Population: hemodialysis patients<br>Sample size: Probiotic (30) +placebo (30)<br>Gender (Male): Probiotic (-) +placebo (-)<br>Mean age (± SD): Probiotic (58.9 ± 8.8) +placebo (60.9 ± 4.4)<br>Inclusive criteria: patients with DN with proteinuria levels>0.3 g per 24 hours, aged 45–85 years.<br>Exclusive criteria: History of active infection within 3 months, intake of probiotic and/or synbiotic supplements within 3 months, history of hospital admission within 3 months, malignancy and/or liver cirrhosis.
Interventions	Treatment group: Probiotics<br>Control group: placebo
Outcomes	Outcomes extracted for meta-analysis:<br>HOMA-IR, serum creatinine, advanced glycation end products (AGEs), BUN, CG, Cockcroft–Gault

	formula to estimate creatinine clearance, fasting plasma glucose(FPG); GSH, total glutathione, homeostasis model of assessment-estimated insulin resistance(HOMA-IR); HbA1c, hs-CRP, malondialdehyde; NO, quantitative insulin sensitivity check index(QUICKI), total antioxidant capacity(TAC), Triglycerides, VLDL-cholesterol, Total cholesterol, LDL-cholesterol, HDL-cholesterol, Total-/HDL-cholesterol ratio, creatinine clearance, Urine protein

12. Miraghajani, M. 2017

Methods	The first author: Miraghajani, M. 2017<br>Study design: RCT (double-blind, placebo-controlled)<br>Study duration: 2 months
Subjects	Country/Region: Iran<br>Population: Type 2 Diabetic Patients with Nephropathy<br>Sample size: Probiotic soy milk (20) + soy milk (20)<br>Gender (Male): Probiotic soy milk (12) + soy milk (10)<br>Mean age (± SD): Probiotic soy milk (56.90 ± 1.81) + soy milk (53.60 ± 1.60)<br>Inclusive criteria: participants with stages 1 and 2 of nephropathy, defined as the meeting criteria including fasting blood glucose > 126 mg/dL, hypoglycemic agents, or insulin intake, proteinuria > 300 mg/day, and glomerular filtration rate > 90 mL/min.<br>Exclusive criteria: changing the dosage of medications, allergy or intolerance to soy milk or avoidance of soy milk consumption, smoking, alcoholism, recent antibiotic therapy, and use of supplements containing vitamins and minerals and any medical condition such as inflammatory bowel disease, infection, liver disease, and rheumatoid arthritis.
Interventions	Treatment group: Probiotic soy milk<br>Control group: soy milk
Outcomes	Outcomes extracted for meta-analysis:<br>The change of MDA, 8-isoPGF2a, GSSG, TAC, GSH, GPx, and GR levels

13. Ramos, C. I. 2019

Methods	The first author: Ramos, C. I. 2019<br>Study design: RCT (double-blind, placebo-controlled)<br>Study duration: 3 months
Subjects	Country/Region: Brazil<br>Population: Nondiabetic, non-dialysis-dependent CKD (NDD-CKD) patients.<br>Sample size: Prebiotic (23) + Placebo (23)<br>Gender (Male): Prebiotic (13) + Placebo (14)<br>Mean age (± SD): Prebiotic (62.2±11.3) + Placebo (52.8±16.1)<br>Inclusive criteria: participants with estimated glomerular filtration rate (eGFR)<45mL/min/1.73m2, aged 18–80 years.<br>Exclusive criteria: Individuals with diabetes mellitus, chronic liver disease, auto-immune disease (i.e. systemic lupus erythematosus, rheumatoid arthritis), congestive heart failure (Stages III/ IV), human immunodeficiency virus, current malignancy, bowel diseases (i.e. inflammatory bowel diseases, celiac disease), cognitive limitations, current smokers and using medications including phosphate binders, immunosuppressants, anti-inflammatories, antibiotics, laxatives, prebiotics, probiotics or synbiotics 3 months preceding baseline assessment.
Interventions	Treatment group: Prebiotic (fructooligosaccharide, FOS)<br>Control group: Placebo
Outcomes	Outcomes extracted for meta-analysis:<br>Primary outcomes: changes in serum (total and free) and urinary (total) PCS. Secondary outcomes: changes in IS, IAA, serum markers of intestinal permeability (zonulin), gut-trophic factors (epidermal growth factor and glucagon-like peptide-2), eGFR, inflammation (high sensitive c-reactive protein and interleukin-6), homeostatic model assessment-insulin resistance, lipid profile and gastrointestinal symptoms.

14. Simeoni, M. 2019

Methods	The first author: Simeoni, M. 2019<br>Study design: RCT (double-blind, placebo-controlled, open-label)<br>Study duration: -
Subjects	Country/Region: Italy<br>Population: Patients with stable CKD stage 3a<br>Sample size: Probiotics (14) + Placebo (14)<br>Gender (Male): Probiotics (9) + Placebo (6)

	Mean age (± SD): Probiotics (61.3 ± 5.2) + Placebo (58.2 ± 6.2)<br>Inclusive criteria: age > 18 years; Caucasian race; Epidemiology Collaboration Equation (EPI) estimated-GFR (eGFR) ranging 60–45 ml/ min/1.73 m2.<br>Exclusive criteria: patients meeting Montreal classification criteria for inflammatory bowel diseases, patients with malabsorption, autoimmune systemic diseases or cancer as well as kidney transplant recipients, pregnant women and patients on current or recent antibiotic therapy or immunosuppressant drugs.
Interventions	Treatment group: Probiotics<br>Control group: Placebo
Outcomes	Outcomes extracted for meta-analysis:<br>The primary outcome: urinary indican and 3-MI concentration<br>The secondary outcomes: concentrations of fecal Lactobacillales and Bifidobacteria and biochemistry laboratory parameters (Iron, TSAT, Ferritin, C-reactive protein, Total cholesterol, HDL cholesterol, Triglycerides, iPTH, Calcium, β2-microglobulin, eGFR.

15. Soleimani 2017

Methods	The first author: Soleimani 2017<br>Study design: RCT (randomized, double-blind, placebo controlled)<br>Study duration: 3 months
Subjects	Country/Region: Iran<br>Population: Diabetic HD patients<br>Sample size: Probiotic (30) + Placebo (30)<br>Gender (Male): Probiotic (20) + Placebo (20)<br>Mean age (± SD): Probiotic (54.0±16.0)+ Placebo (59.4±16.0)<br>Inclusive criteria: Patients aged 18 to 80 years old who were diabetic and who were undergoing regular HD for ≥1 year<br>Exclusive criteria: Pregnant women, individuals with intestinal diseases, those taking probiotic supplements and other forms of probiotics (including probiotic yogurt, kefir, and other fermented foods), those taking prebiotic, antioxidant, and/or anti-inflammatory supplements (e.g., vitamin E, vitamin C, and omega-3 fatty acids), and those taking antibiotics and immunosuppressive medications within 3 months before enrollment in the study.
Interventions	Treatment group: Probiotic<br>Control group: Placebo
Outcomes	Outcomes extracted for meta-analysis:<br>Primary outcome: Parameters of glucose homeostasis.<br>Secondary outcome: lipid profiles, biomarkers of inflammation and oxidative stress, serum creatinine, and BUN concentrations.

16. Soleimani, A. 2019

Methods	The first author: Soleimani, A. 2019<br>Study design: RCT (double-blind, placebo-controlled)<br>Study duration: 3 months
Subjects	Country/Region: Iran<br>Population: Diabetic patients undergoing hemodialysis (HD).<br>Sample size: Synbiotic (30) + Placebo (30)<br>Gender (Male): Synbiotic (21) + Placebo (21)<br>Mean age (± SD): Synbiotic (62.8 ± 12.7) + Placebo (62.8 ± 14.8)<br>Inclusive criteria: Diabetic patients undergoing HD, 18 to 80 years old.<br>Exclusive criteria: Pregnant women, taking probiotic and/or synbiotic supplements, antioxidant and/or anti-inflammatory supplements within the last 3 months prior to the enrollment in the study; patients who required medications adjustment during the study; and those who had recently been diagnosed with T1DM or T2DM.
Interventions	Treatment group: synbiotic capsule *(Lactobacillus acidophilus, Lactobacillus casei, and Bifidobacterium bifidum)*<br>Control group: Placebo
Outcomes	Outcomes extracted for meta-analysis:<br>Primary outcome: the homeostasis model of assessment-insulin resistance (HOMA-IR).<br>Secondary outcomes: Lipid profiles, biomarkers of inflammation, and oxidative stress. (fasting plasma glucose, Insulin, QUICKI, HbA1c, total glutathione, Triglycerides, VLDL-cholesterol, Total cholesterol, LDL-cholesterol, HDL-cholesterol, Total-/HDL-cholesterol ratio, malondialdehyde, hs-CRP, NO, total antioxidant capacity, GSH, subjective global assessment.

17. Guida, B. 2017

Methods	The first author: Guida, B. 2017<br>Study design: RCT (double-blind, placebo-controlled)<br>Study duration: 1 month
Subjects	Country/Region: Italy<br>Population: kidney transplant patients (KTRs)<br>Sample size: Synbiotic (22) + Placebo (12)<br>Gender (Male): Synbiotic (16) + Placebo (12)<br>Mean age (± SD): Synbiotic (54.0±8.9)+ Placebo (47.3±8.5)<br>Inclusive criteria: age > 18 years, transplant vintage > 12 months with stable graft function (serum creatinine < 2.5 mg/dl in the last 3 months), and no episode of acute rejection or infection in the last 3 months.<br>Exclusive criteria: Patients with diarrhea, diabetes, malignancy, pregnancy, food intolerance, autoimmune disorders, severe malnutrition, or clinical conditions requiring artificial feeding.
Interventions	Treatment group: Synbiotic (Probinul Neutro, CadiGroup, Rome)<br>Control group: Placebo
Outcomes	Outcomes extracted for meta-analysis:<br>Total Cholesterol, HDL-Cholesterol, Triglycerides, Glucose, Albumin, eGFR.<br>p-Cresol.

18. Shariaty, Z. 2017

Methods	The first author: Shariaty, Z. 2017<br>Study design: RCT (double-blind, placebo-controlled)<br>Study duration: 1 month
Subjects	Country/Region: Iran<br>Population: The population who suffered from chronic renal failure and underwent hemodialysis.<br>Sample size: probiotic capsules (17) + Placebo (17)<br>Gender (Male): probiotic capsules + Placebo (20)<br>Mean age (± SD): 58<br>Inclusive and Exclusive criteria: Hb levels lower than 110 mg/dL over the past 3 months, being over 17 years in age, undergoing three 4-h dialysis sessions per week, positive CRP results, absence of a severe hyperparathyroidism, absence of active bleeding and surgery in the past 3 months, absence of Hb disorders, absence of anemia due to iron, folate, or vitamin B12 deficiencies, absence of active infections, absence of immune system disorders, absence of malignancies, not being an alcoholic, not being under treatment with antibiotics, not taking corticosteroids and not being infected with HIV.
Interventions	Treatment group: probiotic capsules<br>Control group: Placebo
Outcomes	Outcomes extracted for meta-analysis:<br>Hemoglobin, C-reactive protein

19. Sirich, T. L. 2014

Methods	The first author: Sirich, T. L. 2014<br>Study design: RCT (single-blind)<br>Study duration: 1.5 months
Subjects	Country/Region: Iran<br>Population: patients on hemodialysis.<br>Sample size: resistant starch (20) + digestible starch (20)<br>Gender (Male): resistant starch (11) + digestible starch (13)<br>Mean age (± SD): resistant starch (54±14)+ digestible starch (58±13)<br>Inclusive criteria: aged > 18 years and<br>were stably maintained on outpatient hemodialysis.<br>Exclusive criteria: Patients with a measured residual urea clearance .2 ml/min or if they reported significant urine production, had active gastrointestinal disease, had used antibiotics within 4 weeks, had a record of skipping or shortening their hemodialysis treatments, or if changes in their hemodialysis prescription were planned.
Interventions	Treatment group: resistant starch<br>Control group: digestible starch
Outcomes	Outcomes extracted for meta-analysis:<br>Indoxyl sulfate, p-Cresol sulfate, Urea nitrogen, Albumin, Prealbumin, CRP, Phosphate

20. Viramontes-Hörner, D. 2015

Methods	The first author: Viramontes-Hörner, D. 2015<br>Study design: RCT (double-blind, placebo-controlled)<br>Study duration: 2 months
Subjects	Country/Region: Mexico<br>Population: patients on hemodialysis.<br>Sample size: symbiotic gel (22) + Placebo (20)<br>Gender (Male): symbiotic gel (16) + Placebo (16)<br>Mean age (± SD): symbiotic gel (40.6±17.1)+ Placebo (39.0±16.0)<br>Inclusive criteria: Adult clinically stable hemodialysis patients with arteriovenous fistula, aged 18 years and older, and receiving thrice-weekly hemodialysis for at least 3 months before start the study.<br>Exclusive criteria: usual intake of probiotics, omega-3 fatty acids, pentoxifylline, and immunosuppressive and/or nonsteroidal anti-inflammatory drugs; medical illnesses that affect nutritional and inflammatory status (cancer, decompensated heart failure, chronic liver diseases, intestinal malabsorption, active infections, and acquired immunodeficiency syndrome); filters reuse; and renal transplant antecedent.
Interventions	Treatment group: Symbiotic gel<br>Control group: Placebo
Outcomes	Outcomes extracted for meta-analysis:<br>Albumin, Glucose, Phosphorus, Potassium, Sodium, Calcium, Urea, BUN,<br>Creatinine, CRP, TNF-a, IL-6, Total cholesterol, Triglycerides, c-HDL, c-LDL

21. Mazruei Arani, N. 2019

Methods	The first author: Mazruei Arani, N. 2019<br>Study design: RCT (double-blind, placebo-controlled)<br>Study duration: 3 months
Subjects	Country/Region: Iran<br>Population: patients with DN.<br>Sample size: probiotic (30) + Placebo (30)<br>Gender (Male): —<br>Mean age (± SD): probiotic (62.7±9.1)+ Placebo (60.3±8.5)<br>Inclusive criteria: Participants with DN with a proteinuria level > 0.3 g/24 h, aged 45–85 years old.<br>Exclusive criteria: History of active infection within 3 months, the intake of probiotic and/or synbiotic supplements within 3 months, and malignancy and/or liver cirrhosis.
Interventions	Treatment group: Probiotic<br>Control group: Placebo
Outcomes	Outcomes extracted for meta-analysis:<br>Metabolic profiles, biomarkers of inflammation, and oxidative stress.

22. Xie, L.-M. 2015

Methods	The first author: Xie, L.-M. 2015<br>Study design: RCT (parallel group, placebo-controlled)<br>Study duration: 1.5 months
Subjects	Country/Region: China<br>Population: Hemodialysis patients.<br>Sample size: prebiotic (80) + Placebo (44)<br>Gender (Male): prebiotic (42) + Placebo (26)<br>Mean age (± SD): prebiotic (52.7±14.95)+ Placebo (53.1±13.2)<br>Inclusive criteria: Patients received four-hour HD sessions three times a week with a high-flux membrane dialyzer. The dialysate flow rate was 500 mL/ min and blood flow 250~300 ml/min, Kt/V > 1.2 (1.46 ± 0.13) was achieved.<br>Exclusive criteria: patients with complications with acute inflammation, trauma, gastrointestinal disorders, liver diseases, cancers, mental retardation within recent three months, and patients take supplementation of herbs, antioxidants, vitamins/minerals, and fish oils
Interventions	Treatment group: Prebiotic<br>Control group: Placebo
Outcomes	Outcomes extracted for meta-analysis:<br>Lipid profiles, oxidative and inflammatory status

23. Rossi, M. 2016

Methods	The first author: Rossi, M. 2016<br>Study design: RCT(single–center, double–blind, placebo–controlled, crossover)<br>Study duration: 4 months
Subjects	Country/Region: China<br>Population: Patients with CKD stage 4 or 5.<br>Sample size: Synbiotic (17) + Placebo (20)<br>Gender (Male): Synbiotic (7) + Placebo (14)<br>Mean age (± SD): Synbiotic (68±10)+ Placebo (69±10)<br>Inclusive criteria: Patients with CKD stage 4 or 5 nondialyzed (eGFR=10–30 ml/min per 1.73 m2) ages ≥18 years old.<br>Exclusive criteria: Previous renal transplant; receiving or have received bowel radiation or had large bowel resection; consumed pre- or probiotics or had antibiotic therapy within 1 month of study commencement; medically diagnosed irritable bowel syndrome, Crohn disease, or ulcerative colitis; non-English speaking or unable to give informed consent; likely to receive a transplant or progress to dialysis within 6 months; severely malnourished (Subjective Global Assessment: C); or having had a clinically significant change to their immunosuppressant dose within 6 months (determined by the medical team).
Interventions	Treatment group: Synbiotic<br>Control group: Placebo
Outcomes	Outcomes extracted for meta-analysis:<br>Uremic toxins serum concentrations, kidney related outcomes, inflammatory factors, oxidative stress factors, Gastrointestinal Symptom Score and dietary intake.

24. Khosroshahi 2018

Methods	The first author: Khosroshahi 2018<br>Study design: RCT (double-blind, randomized, parallel, placebo-controlled)<br>Study duration: 2 months
Subjects	Country/Region: Iran<br>Population: Patients with hemodialysis.<br>Sample size: Prebiotic (22) + Placebo (22)<br>Gender (Male): Prebiotic (12) + Placebo (16)<br>Mean age (± SD): Prebiotic (52±11)+ Placebo (60±14)<br>Inclusive criteria: Participants were maintained on chronic hemodialysis thrice weekly for at least six months.<br>Exclusive criteria: Patients who had diabetes, gastrointestinal disease, active inflammatory disorders, infections and malignancy, and patients who had received antibiotic three months prior to enrollment.
Interventions	Treatment group: Prebiotic<br>Control group: Placebo
Outcomes	Outcomes extracted for meta-analysis:<br>Urea nitrogen, creatinine, uric acid, glucose, calcium, phosphorus, alkaline phosphatase lipids, and iron concentrations, TNF-a, interleukin-1b (IL-1b), IL-6, hs-CRP and malondialdehyde (MDA) concentrations, and total anti-oxidant activity, blood urea nitrogen (BUN), serum creatinine, uric acid, lipids, glucose, calcium, phosphorus, alkaline phosphatase, and iron status

25. Khosroshahi 2019

Methods	The first author: Khosroshahi 2019<br>Study design: RCT (double-blind controlled randomized clinical trial)<br>Study duration: 2 months
Subjects	Country/Region: Iran<br>Population: Patients on maintenance hemodialysis<br>Sample size: Prebiotic (25) + Placebo (25)<br>Gender (Male): Prebiotic (14) + Placebo (15)<br>Mean age (± SD): Prebiotic (53.17 ± 10.15) + Placebo (57.90 ± 13.34)<br>Inclusive criteria: Patients who were on maintenance hemodialysis thrice-weekly for at least 6months and had at least 18 years of age.<br>Exclusive criteria: Patients who had diabetes, gastrointestinal diseases, active inflammatory disorders, infections, malignancies, changes in dialysis planning or pattern, or those who have received antibiotics prior to the enrollment.
Interventions	Treatment group: Prebiotic<br>Control group: Placebo

Outcomes	Outcomes extracted for meta-analysis:<br>serum urea nitrogen and creatinine and uric acid, Hb, Hct, ferritin, TIBC, intact PTH, total cholesterol, HDL cholesterol or triglyceride, p-cresol, hs-CRP, IS, TAC.

26. Laffin 2019

Methods	The first author: Laffin 2019<br>Study design: RCT (double-blind, parallel, randomized, placebo-controlled)<br>Study duration: 2 months
Subjects	Country/Region: Iran<br>Population: ESRD patients undergoing hemodialysis.<br>Sample size: Prebiotic (9) + Placebo (11)<br>Gender (Male): Prebiotic (6) + Placebo (7)<br>Mean age (± SD): Prebiotic (53.8±11.8)+ Placebo (57.6±9)<br>Inclusive criteria: ESRD patients undergoing hemodialysis.<br>Exclusive criteria: Individuals with gastrointestinal disease, diabetes, active inflammatory disorders, infections, and malignancy and patients who had been treated with antibiotics within 3 months before the enrollment in the study.
Interventions	Treatment group: Prebiotic<br>Control group: Placebo
Outcomes	Outcomes extracted for meta-analysis:<br>Urea nitrogen, uric acid, calcium, phosphorus, and parathyroid hormone, TNFα and IL-6, malondialdehyde

27. Mirzaeian 2019

Methods	The first author: Mirzaeian 2019<br>Study design: RCT (double-blind placebo-controlled randomized)<br>Study duration: 2 months
Subjects	Country/Region: Iran<br>Population: Patients on hemodialysis.<br>Sample size: Synbiotic (21) + Placebo (21)<br>Gender (Male): Synbiotic (14) + Placebo (16)<br>Mean age (± SD): Synbiotic (58.30±11.3) + Placebo (69.74±42.87)<br>Inclusive criteria: Patients performing hemodialysis treatment 3-times per week for at least 4 hours each time, not being pregnant or lactating, no history of active cancers, no history of severe chronic conditions such as pulmonary, cardiovascular, and hepatic diseases, no addiction to alcohol or drugs, and lack of gastrointestinal disorders, HIV disease, and psychiatric problems.<br>Exclusive criteria: Patients with severe edema, suffering from infections during 4 past weeks, and those who used synbiotic, probiotic, prebiotic or antibiotics during 4 weeks preceding the study, immune-suppressive medicines, and anticoagulant agents. Patients were not willing to continue the study, did not respond to the questions completely, and reported any side effects due to synbiotic consumption.
Interventions	Treatment group: Synbiotic<br>Control group: Placebo
Outcomes	Outcomes extracted for meta-analysis:<br>The primary outcomes: uremic toxins (Indoxyl sulfate and phenol).<br>The secondary outcomes: liver enzymes, blood urea nitrogen and creatinine, high sensitive C-reactive protein, some minerals, parathyroid hormones, albumin, and blood pressure.

28. Dehghani 2016

Methods	The first author: Dehghani 2016<br>Study design: RCT (randomized controlled double blinded clinical trial)<br>Study duration: 1.5 months
Subjects	Country/Region: Iran<br>Population: patients with CKD (stages 3 and 4).<br>Sample size: Synbiotic (31) + Placebo (35)<br>Gender (Male): Synbiotic (23) + Placebo (27)<br>Mean age (± SD): Synbiotic (63.00 ± 6.52) + Placebo (60.00 ± 8.33)<br>Inclusive criteria: Age between 35 and 75 years and suffering from CKD stage 3 or 4 (glomerular filtration rate [GFR], 15 mL/min/1.73 m2 to 59 mL/min/1.73 m2).<br>Exclusive criteria: Pregnancy in women, use of antibiotics and lactulose 14 days before the start of the study, alcohol dependence, and hepatitis or HIV infection. Attrition criteria were the use of antibiotics and lactulose during the study and starting treatment with hemodialysis.
Interventions	Treatment group: Synbiotic

	Control group: Placebo
Outcomes	Outcomes extracted for meta-analysis:<br>Blood urea nitrogen, uric acid, and creatinine of serum and urine, the 24-hour urine creatinine clearance and GFR
